# Mechanism of AF9 (MLLT3)–Partner Dissociation in Mixed-Lineage Leukemia-Rearranged Leukemia

**DOI:** 10.1021/jacsau.5c01454

**Published:** 2026-03-07

**Authors:** Shilpa Sharma, Keya Joshi, Arjun Saha

**Affiliations:** † Department of Chemistry and Biochemistry, 118733 University of Wisconsin-Milwaukee, Milwaukee, Wisconsin 53211, United States; ‡ Computational Medicine and Pharmacology, 2331 University of North Carolina, Chapel Hill, North Carolina 27599, United States; § Department of Computational Biology and Bioinformatics, 2331 University of North Carolina, Chapel Hill, North Carolina 27599, United States

**Keywords:** MLL-rearranged leukemia, PPI-GaMD simulations, AF9, free-energy landscape, peptide dissociation, enhanced sampling, intrinsically disordered proteins

## Abstract

Chromosomal rearrangements involving the mixed-lineage leukemia (MLL) gene drive aggressive leukemias with a poor prognosis. AF9 (MLLT3), a YEATS family protein, is a core component of transcriptional and epigenetic regulatory complexes essential for hematopoietic stem cell maintenance. In MLL-rearranged leukemia, the MLL–AF9 fusion protein aberrantly recruits transcriptional and epigenetic modifiers, including DOT1L, BCOR, and CBX8, disrupting normal hematopoietic gene regulation. Despite the importance of these interactions, the molecular mechanisms underlying AF9–partner(s) binding and their dissociation remain unclear. Here, we employed **P**rotein–**P**rotein **I**nteraction–**G**aussian **a**ccelerated **M**olecular **D**ynamics (PPI–GaMD) simulations to probe the dissociation pathways of AF9–bound peptides. Free-energy landscapes revealed that the dissociation process proceeds in a stepwise manner through metastable intermediates. Dissociation predominantly occurred through channel 1, a broad electrostatically asymmetric face of AF9. Consistent with the experimental findings, DOT1L formed the most stable complex with AF9, followed by CBX8 and then BCOR. Distinct intermediate conformations and interaction patterns were observed for each partner, reflecting their differential binding stabilities. Partner release was primarily driven by electrostatic interactions, while metastable intermediates were stabilized by hydrophobic contacts. The extended hydrophobic surface of DOT1L accounted for its enhanced binding, evident from its dominant van der Waals contribution. Clustering analysis identified dominant intermediate conformations that highlight critical steps in peptide dissociation and provide structural templates for inhibitor design. These metastable states represent druggable conformations that can be leveraged in structure-based screening, offering a foundation for targeted therapies in MLL-rearranged leukemias.

## Graphical abstract

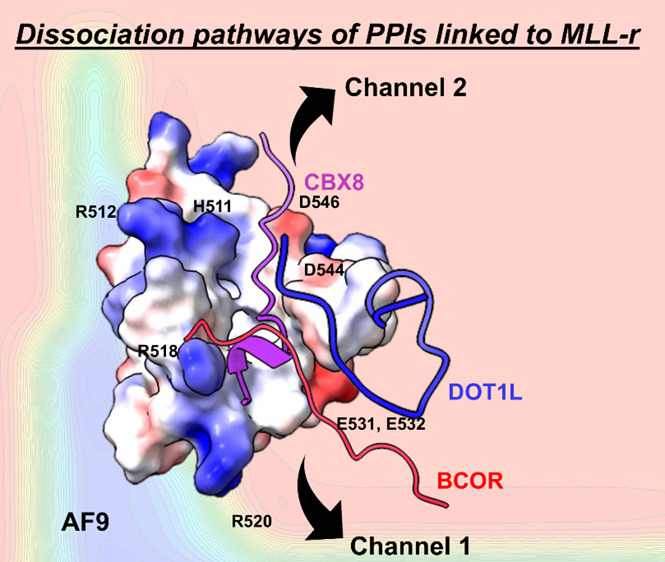

## 1 Introduction

The mixed lineage leukemia (MLL; KMT2A) gene regulates the transcription of numerous target genes and is essential for H3K4 trimethylation and transcriptional initiation at a subset of HOX promoters.^1^ Rearrangements involving the MLL gene define a distinct and aggressive subtype of acute leukemia that can manifest as either acute myeloid leukemia (AML) or acute lymphoblastic leukemia (ALL).^2^ These translocations generate oncogenic MLL fusion proteins that aberrantly activate HOX gene clusters and MEIS1, thereby sustaining self-renewal and blocking hematopoietic differentiation. Clinically, MLL-rearranged (MLL-r) leukemias exhibit a poor prognosis, with high relapse rates and limited responsiveness to conventional chemotherapy, underscoring the urgent need for targeted therapeutic strategies.^2^ Transcriptional activation by MLL fusions is mediated by the recruitment of the AEP (AF4/ENL/P-TEFb) and DotCom (DOT1L–AF10/ENL) complexes.^3,4^ Among over 90 known MLL fusion partners, nearly 70% involve AEP components, with ∼34% of MLL-r leukemias harboring the MLL–AF9 fusion, underscoring its clinical significance.^5^

AF9 (MLLT3), a YEATS family protein, is a key component of transcriptional and epigenetic regulatory complexes and is critical for maintaining hematopoietic stem and progenitor cells.^6,7^ Its C-terminal ANC1 homology domain (AHD) fuses with the MLL N-terminus, enabling interactions with transcriptional activators such as AF4 family members and DOT1L, as well as transcriptional repressors including BCOR and CBX8.^4,8–10^ AF4 promotes P-TEFb–dependent phosphorylation of RNA polymerase II and DOT1L methylates H3K79 to promote transcription, whereas BCOR and CBX8 mediate gene silencing via polycomb-associated chromatin compaction. Fusion with MLL deregulates AF9 recruitment, leading to persistent activation of target genes and oncogenic transformation of hematopoietic cells, making these protein–protein interactions (PPIs) central to MLL–AF9 leukemia pathogenesis.^11^

Bushweller and colleagues^8–11^ have made seminal contributions to elucidating the structural and functional mechanisms governing AF9 and its interactions. Circular dichroism (CD) experiments revealed that AF9-AHD possesses minimal secondary structure with limited β-content.^10^ In contrast, our previous replica exchange molecular dynamics (REMD) simulations demonstrated that when BCOR is removed from the AF9–BCOR NMR complex, AF9 rapidly loses its β-sheet structure, while α-helices remain stable throughout the simulation.^12^ AF9, AF4, DOT1L, BCOR, and CBX8 contain extensive intrinsically disordered regions (IDRs) that lack stable tertiary structure under physiological conditions.^13^ The structural flexibility of IDRs enables them to engage in multivalent and dynamic interactions^14^ but also complicates the experimental characterization of the full-length complexes. Therefore, short peptide constructs encompassing the core binding motifs offer a biophysically tractable means of investigating the fundamental interaction patterns that underlie partner recognition and dissociation. Bushweller and co-workers identified the minimal interacting regions of three AF9 partners and determined NMR structures of AF9 in complex with the corresponding peptide partners, including DOT1L (PDB: 2MV7 ^9^), BCOR (PDB: 6B7G^8^), and CBX8 (PDB: 2N4Q^8^); their structures are shown in Figure 1. The authors have also provided a complex structure of the AF9–AF4 complex, where AF4 is covalently bound to AF9 (PDB: 2LM0 ^10^). These complexes reveal a conserved binding mechanism in which a β-strand of alternating hydrophobic residues from the partner forms an antiparallel β-sheet with AF9’s β-hairpin, while three surrounding α-helices of AF9 wrap around the interface, burying hydrophobic residues and stabilizing the complex. Collectively, these studies established that AF9 undergoes a folding-upon-binding mechanism across all partners, using a common and mutually exclusive binding interface.^11^ Notably, NMR relaxation data indicate that AF9 retains intrinsic flexibility even in the bound state, suggesting dynamic exchange among disordered partners; however, the precise mechanism governing this partner exchange remains unresolved.^11^

**Figure 1:**
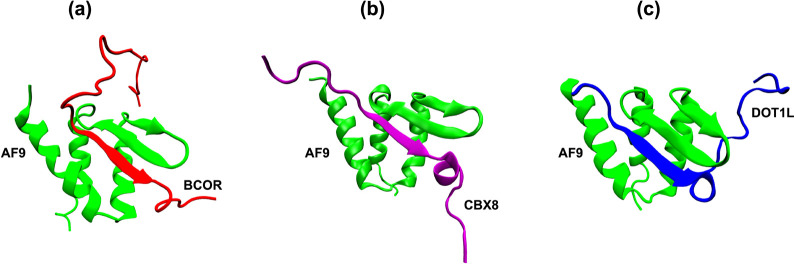
NMR structures of AF9 interacting with its binding partners: (a) BCOR, (b) CBX8, and (c) DOT1L.

Experimental *K*_D_ values further reveal a hierarchy of AF9–partner affinities—DOT1L (4 nM) binds most tightly, followed by extended BCOR (18 nM), whereas CBX8 (>500 nM) and the shorter BCOR peptide (>2000 nM) exhibit markedly weaker interactions.^8^ Schmidt et al.^8^ identified the 53-residue *extended BCOR* segment (residues 1176–1228) as the high-affinity AF9-binding region, stabilized by electrostatic interactions involving AF9 residue E531. However, due to NMR limitations, the structure was determined using a shorter BCOR peptide (residues 1176–1207), as the C-terminal region exhibited severe line broadening from conformational exchange. Importantly, the shorter construct produced well-dispersed NMR spectra with complete resonance assignments, enabling a high-resolution structural characterization of the complex. Structure-guided mutagenesis of AF9 provided insight into how specific interactions contribute to leukemogenesis. For instance, in the AF9–DOT1L complex, D544R and D546R mutations weaken DOT1L binding to MLL-AF9, leading to a graded loss of DOT1L recruitment, differential reduction of H3K79 methylation at target loci, and diminished leukemic transformation potential.^9^ Similarly, Schmidt et al.^8^ demonstrated that the E531R AF9 mutation selectively disrupts BCOR binding and abolishes leukemogenic activity, notably downregulating *EYA1*, one of the key AF9–BCOR target genes. Nikolovska-Coleska and co-workers^15^ further validated the AF9–DOT1L PPI as a therapeutically tractable target in MLL-rearranged leukemia. Several independent studies (Li et al.,^16^ Wu et al.,^17^ and Mishra et al.^18^) have reported the synthesis and characterization of small-molecule inhibitors that disrupt the AF9–AF4/DOT1L interactions. In particular, Mishra et al.^18^ synthesized and evaluated a library of 67 quinoxaline-based compounds and identified inhibitors with IC_50_ values in the sub- to low-micromolar range (0.35–1.5 μM), underscoring the druggability of these PPI interfaces for small-molecule inhibitor development. Understanding the molecular basis of their differential binding and dissociation dynamics is therefore crucial for designing selective inhibitors that can modulate AF9’s interaction network and advance therapeutic strategies against MLL-r leukemia.

Capturing protein–protein or protein–peptide dissociation events at atomistic resolution poses a major challenge for conventional molecular dynamics (cMD) simulations, as these processes typically occur on microsecond to millisecond time scales and are also due to the extremely high flexibility of the peptides.^19^ To overcome these limitations, various enhanced sampling methods—such as umbrella sampling^20^ and metadynamics^21^—have been developed. However, these approaches generally require the prior identification of suitable collective variables (CVs) to describe the reaction coordinate. To address this limitation, Wang and co-workers introduced the PPI Gaussian accelerated molecular dynamics (PPI-GaMD) method,^22^ which enables the direct observation of reversible binding and dissociation events without predefined CVs. PPI-GaMD selectively boosts the interaction potential energy between protein partners to accelerate slow dissociation events, while an additional boost potential applied to the remaining system potential energy enhances protein flexibility and promotes reassociation. Using this approach, Wang et al. successfully captured multiple binding pathways in the barnase–barstar system and obtained binding free energies and kinetic parameters in agreement with experimental measurements.^22^ More recently, Keya et al. employed PPI-GaMD to investigate peptide agonist dissociation in adhesion G protein–coupled receptors (ADGRs), further demonstrating its applicability to complex biomolecular systems.^23^

In our previous study,^12^ we developed the Mix and Match (M&M) method to map the binding free-energy landscape (BFEL) of the AF9–BCOR complex. By combining enhanced sampling simulations with rigorous free energy calculations, we identified multiple low-energy conformations beyond those observed in the NMR structure.^8^ These analyses revealed additional stabilizing interactions between the C-terminal residues of BCOR and AF9, which enhanced the overall binding affinity. Building on this foundation, the present study moves beyond the static characterization of the binding landscape to explore the dynamic dissociation mechanisms of AF9 with its key peptide partners—BCOR, CBX8, and DOT1L—using the PPI-GaMD approach. Because these simulations focus on the core interaction motifs, peptide fragments of BCOR, DOT1L, and CBX8 were used as representative models instead of the full-length proteins. To close this gap in understanding, we focused on answering the following central questions: How do these three peptides of equal length (minimally interacting regions) dissociate differently from AF9? What role does their sequence composition play in modulating their differential binding behavior? What are the prominent intermolecular interactions stabilizing each complex? What distinct low-energy conformational states emerge during the dissociation process? While our earlier work delineated the thermodynamics of complex formation, here we focus on the mechanisms of dissociation, identifying transient intermediates, key interfacial contacts, and potential allosteric pathways that modulate AF9–partner stability. This integrated understanding of dissociation provides mechanistic insight into AF9 regulation and offers a rational basis for structure-guided design of inhibitors targeting AF9-mediated PPIs implicated in MLL-rearranged leukemia. Although AF4 is a well-established binding partner of AF9, this study focuses on AF9 complexes with noncovalently bound peptide partners for which stable dimeric structures suitable for dissociation simulations are available. Because PPI-GaMD is designed to capture reversible noncovalent unbinding events, AF9–AF4 interactions involving covalent attachment were not considered.

To this end, five independent 500 ns PPI-GaMD simulations were performed for each AF9–peptide (BCOR, CBX8, and DOT1L) complex, providing a total sampling time of 2.5 μs per system. The resulting trajectories enabled the characterization of dissociation pathways and free-energy landscapes (FELs), offering atomistic insights into the interplay of hydrophobic and electrostatic interactions governing AF9–partner recognition. Furthermore, we employed the MM-GBSA^24^ approach to estimate binding free energies and assess their consistency with experimental observations.

## 2 Results and Discussion

### 2.1 MM-GBSA Analysis Reveals Differential Binding Affinities of BCOR, CBX8, and DOT1L Peptides to AF9

To investigate the mechanistic binding pathways of AF9 with its partners, we first assessed their relative binding strengths using MM-GBSA^24^ calculations. The analysis revealed distinct differences in the contributions of individual residues to the complex stability. Across all AF9 complexes, the total binding free energy was predominantly driven by van der Waals and electrostatic interactions, supplemented by a modest favorable contribution from nonpolar solvation energy (Figure 2, Table S1). In contrast, the polar solvation energy imposed a substantial energetic penalty on binding. Among the peptide partners, DOT1L (residues 876–900) exhibited the most favorable overall binding free energy (−113 kcal/mol) with AF9, followed by CBX8 (residues 326–349; −94.5 kcal/mol), and BCOR (residues 1175–1207; −84.7 kcal/mol), consistent with experimental evidence indicating that AF9 binds most tightly to DOT1L, with intermediate affinity to CBX8, and weakest to BCOR. Among the three complexes, DOT1L exhibited the strongest electrostatic contribution to binding, although this is largely counterbalanced by the substantial penalty from polar solvation energy. Entropic contributions were not explicitly included in the MM-GBSA binding free energy estimates. For protein–peptide systems such as the AF9 complexes studied here, accurate entropy estimation is particularly challenging due to the pronounced conformational flexibility of both binding partners. Under these conditions, commonly used normal-mode and quasiharmonic approaches are known to be poorly converged and may introduce substantial noise. Because all complexes examined involve the same protein and closely related binding poses, entropic contributions are expected to partially cancel in relative comparisons. While absolute MM-GBSA binding free energies cannot be directly converted into experimental dissociation constants due to inherent methodological approximations, the calculations are intended to provide a qualitative comparative assessment of binding. In particular, the relative ranking of MM-GBSA Δ*G* values and the residue-level energy decomposition offer a useful framework for rationalizing differences in AF9 binding to its partners, as summarized in Table 1.

**Table 1:** Calculated Binding Free Energies of AF9 Complexes from MM-GBSA Method, Along with Their Experimental *K*_D_ Values^11^

**s. no.**	**system**	**MM-GBSA energy** (kcal/mol)	**experimental** *K*_**D**_ **(nM)**
1	AF9–BCOR	–84.7	>2000
2	AF9–CBX8	–94.5	>500
3	AF9–DOT1L	–113	>4 ± 1

**Figure 2:**
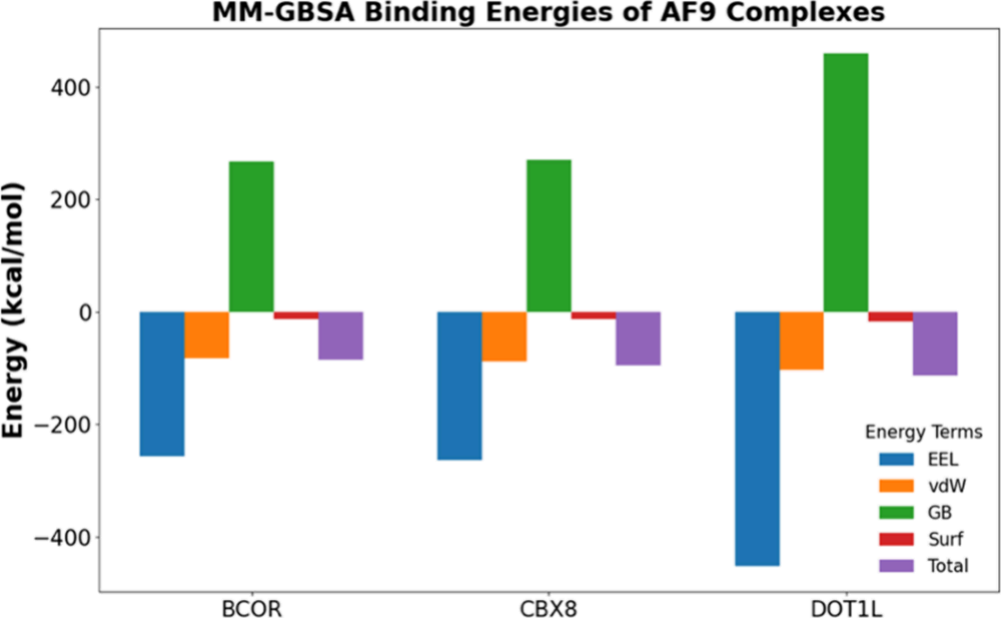
Binding free energies and energy components obtained from MM-GBSA analysis of AF9–BCOR, AF9–CBX8, and AF9–DOT1L complexes. Here, EEL, vdW, GB, and Surf denote the electrostatic, van der Waals, polar solvation, and nonpolar solvation energy contributions to the total binding free energy, respectively.

Per-residue energy decomposition analysis revealed that AF9 residues H511, M515, L523, T542, F543, F545, L547, and C548 contributed most significantly to binding through van der Waals interactions, whereas R520, D544, and D546 contributed primarily via electrostatic interactions (Tables S2–S4). In the AF9–BCOR complex, the key interacting residues of BCOR were identified as N1193, L1194, K1195, V1196, I1198, L1200, and L1203 (Table S2). For the AF9–CBX8 complex, residues L333, I334, A335, R336, I337, P338, and V339 of CBX8 contributed more substantially, consistent with its intermediate binding affinity (Table S3). In the AF9–DOT1L complex, the extensive hydrophobic surface of DOT1L, comprising residues L879, K878, P880, V881, S882, I883, L885, L890, and P891, made the largest contribution to the binding energy, accounting for its highest overall affinity (Table S4).

To quantitatively assess sequence-dependent hydrophobic contributions, hydrophobic energies were computed as the sum of van der Waals and nonpolar solvation terms for interfacial hydrophobic residues from both AF9 and the peptide. As shown in Table 2, the total hydrophobic stabilization is strongest for the AF9–DOT1L complex (−66.2 kcal/mol), while comparable values were observed for AF9–BCOR (−55.16 kcal/mol) and AF9–CBX8 (−53.35 kcal/mol). Decomposition of the hydrophobic energy reveals that DOT1L contributes a substantially larger hydrophobic component from the peptide side, consistent with its more hydrophobic sequence and tighter packing against the AF9 binding groove.

**Table 2:** Hydrophobic Energy Contributions of Interfacial Residues from AF9 and Its Peptide Partners, Together with the Buried Nonpolar Surface Area of the AF9–Peptide Complexes

s. no.	complex	*E*_Hydrophobic_ (total, kcal/mol)	*E*_Hydrophobic_ (AF9, kcal/mol)	*E*_Hydrophobic_ (Pep, kcal/mol)	buried nonpolar surface area (nm^2^)
1	AF9–BCOR	–55.16	–22.51	–32.65	9.69
2	AF9–CBX8	–53.35	–20.87	–32.48	10.76
3	AF9–DOT1L	–66.2	–26.5	–39.70	12.96

An independent structural metric, buried nonpolar surface area, was calculated for each complex to correlate with the observed hydrophobic energy. The buried nonpolar surface area upon binding increases systematically from BCOR (9.69 nm^2^) to CBX8 (10.76 nm^2^) to DOT1L (12.96 nm^2^), which further supports the trend. The larger buried hydrophobic surface in the AF9–DOT1L complex indicates more extensive nonpolar burial at the interface, providing quantitative evidence that hydrophobic interactions contribute favorably to binding and follow the observed hierarchy (BCOR < CBX8 < DOT1L).

### 2.2 PPI-GaMD Simulations of AF9–Partner Complexes

PPI-GaMD simulations^22^ were carried out on all three AF9 complexes to elucidate the dissociation mechanisms of peptides of equal length. Center of mass distances, native contacts between protein and peptide, and Peptide root-mean-square deviation (RMSD), and the distance between key interaction-forming residues were chosen as primary reaction coordinate pairs for constructing 2D FELs. Interaction analyses based on NMR structures of AF9–peptide complexes identified F545–V1196, F543–I337, and F543–I883 as the dominant residue pairs in AF9–BCOR, AF9–CBX8, and AF9–DOT1L, which were monitored and incorporated as reaction coordinates to capture the free energy profiles of peptide dissociation.

#### 2.2.1 Two Alternative Pathways Were Observed for BCOR Peptide Dissociation from AF9

In five independent 500 ns PPI-GaMD production simulations,^22^ the complete dissociation of BCOR from the binding pocket of AF9 was observed within 100 ns as the RMSD of the peptide increased to ≥30 Å (Figure S1a). Across the five simulations, the boost potentials were consistent, with average values ranging from ∼9 to 10.39 kcal/mol and standard deviations (SDs) between ∼4.59 and 5.34 kcal/mol (Table S8). We then combined all PPI-GaMD production runs to generate the combined free energy profile for BCOR dissociation from AF9 using energetic reweighting (see Section 4).

The center-of-mass (COM) distance and the number of native contacts between AF9 and BCOR were selected as reaction coordinates for the construction of the 2D FEL of the AF9–BCOR complex. The average COM distance (Figure 3a) was observed to increase after ∼50 ns, exceeding 20 Å and continuing to rise until ∼100 ns, at which point BCOR was fully dissociated from AF9. Similar trends were observed for the RMSD of BCOR and the F545–V1196 inter-residue distance across all five simulations (Figure S1a,b). The number of native contacts was also found to decrease sharply after ∼20 ns and approached zero by 100 ns, consistent with the complete dissociation of BCOR (Figure 3b). In all five simulations, complete dissociation of BCOR occurred within 100 ns (Figure S1). Subsequently, the simulations were extended to 500 ns to verify the stability of the fully dissociated state, exclude reassociation events, and assess conformational changes in AF9 following peptide release.

**Figure 3:**
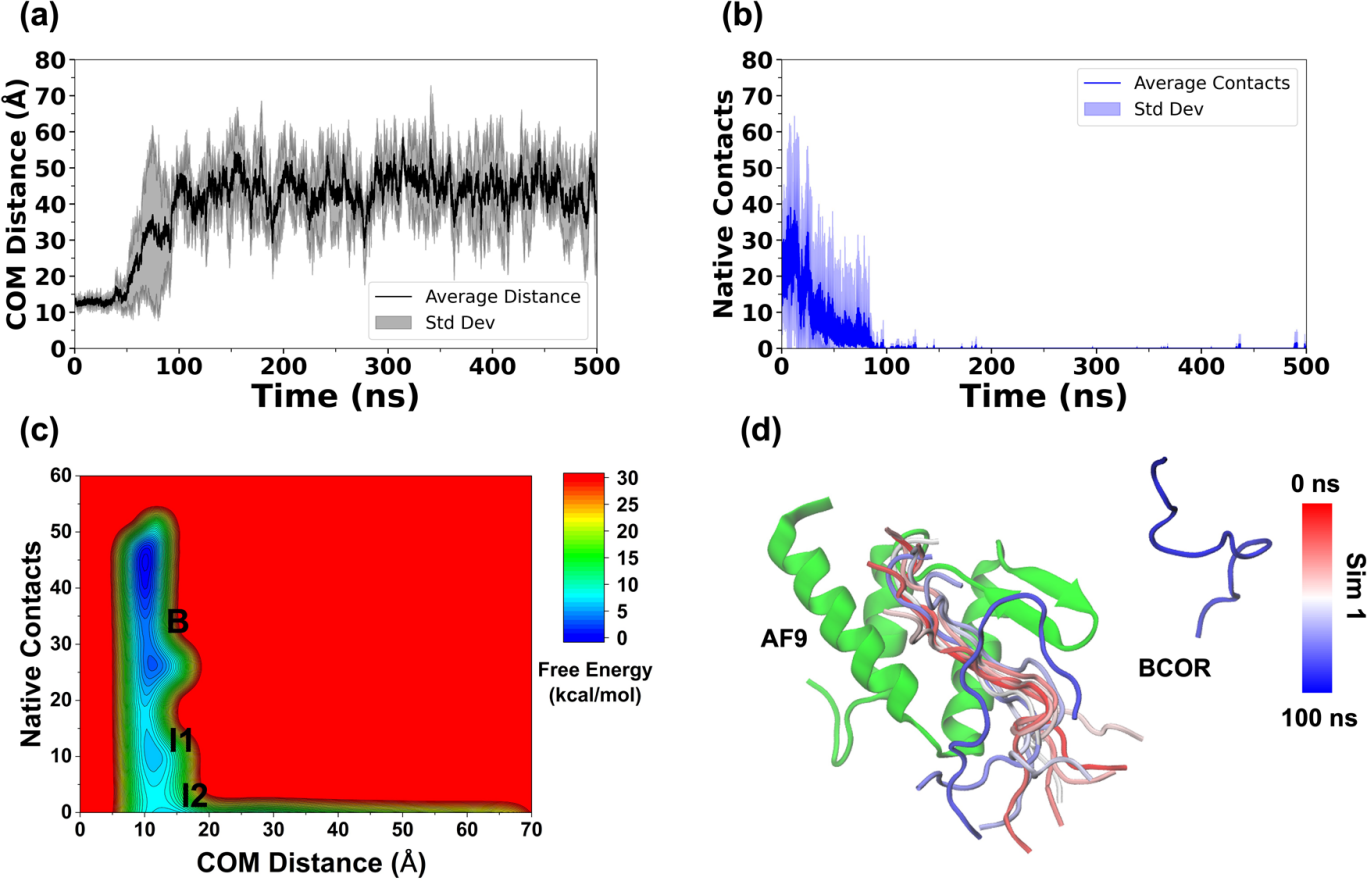
Plots of (a) average COM distance, (b) native contacts between AF9 and BCOR, and (c) combined free-energy landscape of BCOR dissociation from AF9. The low-energy states are labeled as “Bound” (B), “Intermediate 1” (I1), “Intermediate 2” (I2), and (d) the dissociation pathway of BCOR in Simulation 1.

The dissociation of BCOR was carefully examined across the five independent trajectories, and individual 2D FELs were constructed for each case (Figures S2 and S3). In the individual 2D FEL, constructed as a function of COM distance and native contacts, a broad lower energy basin was observed in Sim 1, 3, and 5, suggesting the presence of bound and intermediate conformations (Figure S2a,c,e). A distinct, less populated energy basin was observed in Sim 2, 3, and 4, suggesting the presence of another intermediate species (Figure S2b–d). The combined 2D-FEL of the AF9–BCOR complex exhibited three distinct low-energy states identified as Bound (B), Intermediate 1 (I1), and Intermediate 2 (I2) (Figure 3c). The COM distance and native contacts between AF9 and BCOR for each of the states (Bound, Intermediate 1, and Intermediate 2) were centered at (11.2 Å, 27), (11.4 Å, 9), and (12.7 Å, 0), respectively.

The FELs constructed by using native contacts are well-suited for distinguishing bound and intermediate conformations in which interfacial contacts are retained. However, once native contacts are completely lost, these CVs collapse a wide range of dissociated configurations into a single region. To better resolve the structural diversity of fully dissociated states, additional two-dimensional FELs were therefore constructed using the peptide RMSD and the F545–V1196 inter-residue distance (Figure S3). Across all simulations of the AF9–BCOR complex, a well-defined energy minimum corresponding to the bound state was consistently observed, accompanied by at least one intermediate state. While Sim 1–3 exhibited similar energy basins, Sim 4 and Sim 5 revealed multiple intermediate states at higher RMSD values. The combined 2D FEL of the AF9–BCOR complex revealed a bound state (B), an intermediate state (I3) characterized by higher RMSD and inter-residue distance values, and an unbound state (U) (Figure S3f). Notably, the I3 state was not resolved in the 2D-FEL constructed using the center-of-mass distance and native contacts (Figure 3c) and likely corresponds to a late intermediate sampled immediately prior to complete dissociation. The BCOR RMSD and F545–V1196 inter-residue distance values corresponding to the bound, late-intermediate (I3), and unbound states were centered at (3.48 Å, 6.23 Å), (36.10 Å, 24.41 Å), and (67.17 Å, 47.97 Å), respectively.

DPeak clustering^25^ was performed on the concatenated trajectories from the five simulations, and representative complex structures corresponding to the four distinct energy states, identified in the FEL (Figures 3, S2, and S3), were obtained. A total of 33 clusters were obtained, with the most populated cluster corresponding to the unbound state. The clusters, their populations, and representative frames are summarized in Table S9. Based on structural descriptors including native contacts and center-of-mass distance, the 33 clusters were classified into bound, intermediate, and unbound states, comprising 6, 9, and 18 clusters, respectively. Cluster 8 (C8) was assigned to the bound state, while clusters 2, 11, and 20 (C2, C11, C20) were assigned to intermediates I1, I2, and I3, respectively, by carefully mapping them to the combined FEL, and their representative structures and molecular interactions were visualized (Figure 4).

**Figure 4:**
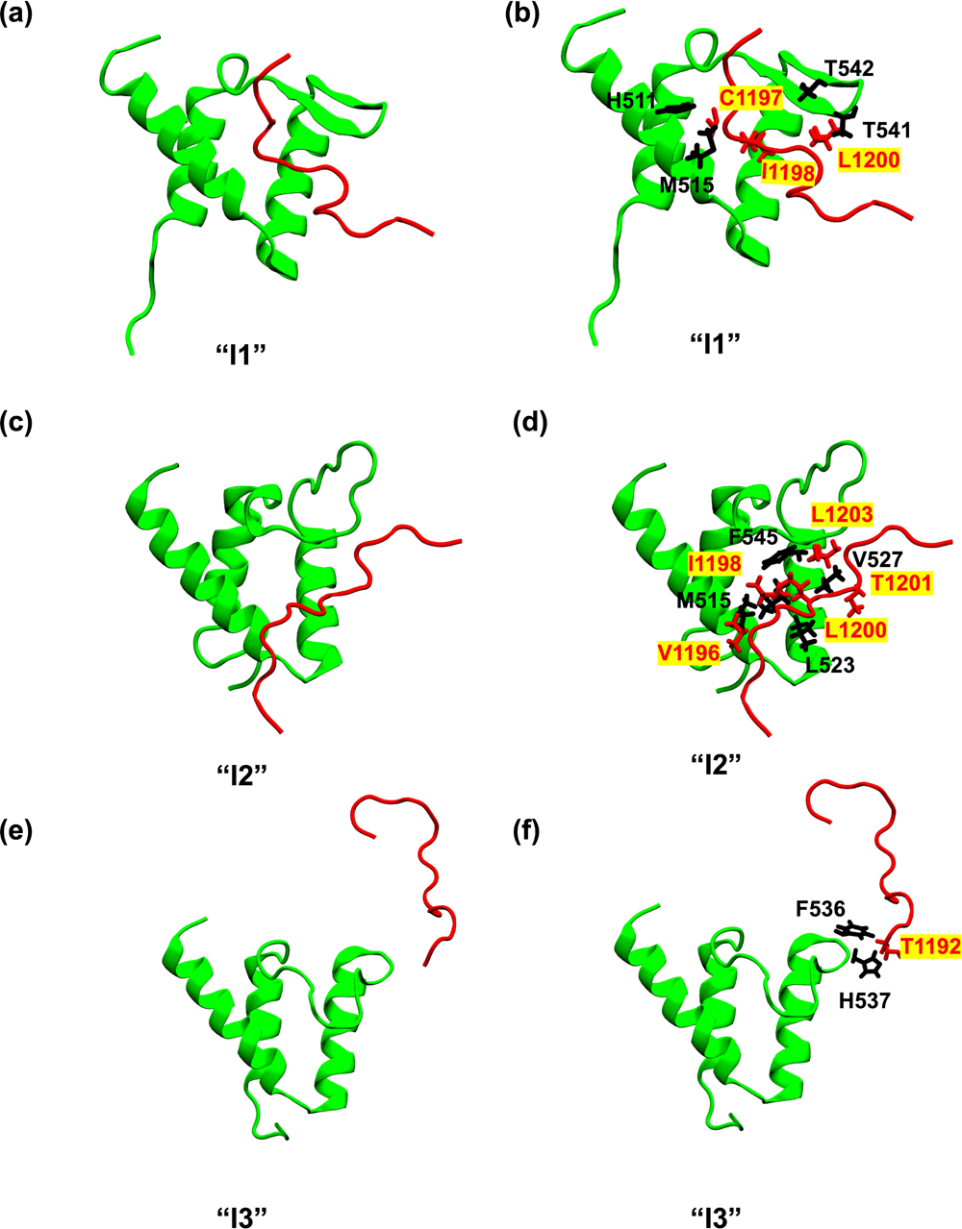
Representative structures and associated molecular interactions for the intermediate states I1 (a, b), I2 (c, d), and I3 (e, f). The intermediate states were obtained from the DPeak clustering analysis and were carefully mapped to the free-energy landscape of AF9–BCOR dissociation.

The bound and intermediate states were characterized by examining the dominant interactions between AF9 and BCOR. In the bound state (B), the interactions observed in the NMR structure^8^ were preserved, particularly those contributing most strongly to the binding free energy, such as F545–V1196. In I1, BCOR shifted away from the binding pocket, completely losing the F543–V1196 interaction but retaining F543–I1198. Additional hydrophobic interactions emerged in I1, notably H511–C1197, M515–C1197, T541–I1198, T542–L1200, and I538–L1200 (Figure 4a,b). In the I2 intermediate state, BCOR lost all native contacts with AF9, while forming alternative hydrophobic interactions involving M515–V1196, M515–I1198, F545–L1200, F545–L1203, and V527–G1202 (Figure 4c,d). Notably, in this conformation, the C-terminal region of BCOR was observed to bind within an adjacent cavity that becomes accessible following a complete loss of AF9 β-sheet structure, suggesting a distinct binding mode that requires further investigation. In I3, all native contacts disappeared, and new non-native interactions were observed, including F536–T1192 and H537–T1192 (Figure 4e,f). The smaller population of C20 suggested that I3 is a transient non-native state that precedes complete unbinding, and not all dissociations necessarily go through it. Across the bound and intermediate states, hydrophobic contacts were the dominant contributors to the AF9–BCOR interactions. Two-dimensional interaction maps for these states were also generated using MOE^26^ (Figure S4) to clearly visualize residues involved in hydrophobic interactions.

Structural changes in AF9 were also monitored during BCOR dissociation. Comparison of AF9 in bound, intermediate, and unbound states revealed that the β1 and β2 strands shifted closer to α1 in the intermediate states. Upon complete BCOR dissociation, β1 and β2 collapsed toward α1, resulting in the loss of the β-sheet structure and the collapse of the binding pocket (Figure S5). These results suggest that BCOR dissociates via a stepwise mechanism initiated by disruption of key N-terminal contacts, stabilization of non-native hydrophobic intermediates, and eventual collapse of the AF9 binding pocket.

To gain deeper insights into the dissociation mechanism of the BCOR peptide from AF9, trajectories from the five independent PPI-GaMD simulations were closely analyzed. Two probable dissociation channels of AF9 are illustrated in Figure S6. In channel 1, the C-terminal region of the peptide engaged with AF9, whereas channel 2 accommodated the N-terminal region. Channel 1 was primarily formed by residues from α1, α2, and β2. The loop connecting α1 and α2, together with adjacent residues, created a positively charged patch through residues R518 and R520. In contrast, residues D544 and T541–T542 from β2 contributed a negatively charged patch that stabilized peptide binding. Additionally, a strongly negatively charged cavity was located adjacent to the C-terminal binding site, formed by β1, β2, and α2, with residues E531–E532 of α2 contributing further negative potential that may influence peptide dissociation (Figure S6b). Channel 2 was defined by α1, α3, and β2. Compared with channel 1, it was shallower and lacked a deep binding pocket. Within this channel, R512 and H511 of α1 generated a positive patch, while D546 of β2 provided a negatively charged anchor point for the BCOR peptide (Figure S6). Thus, both channels exhibited marked electrostatic asymmetry, and peptide dissociation could proceed through either pathway.

Our simulations revealed two distinct dissociation pathways for BCOR from channel 1 of AF9 (Figure 5). In some trajectories, BCOR dissociated directly from the canonical binding pocket into the solvent (direct dissociation pathway), (Figure 5a,d). In others, BCOR first transitioned into an adjacent cavity, forming a metastable intermediate, before ultimately dissociating into the solvent (cavity-assisted dissociation pathway) (Figure 5b,e). Direct dissociation from channel 1 was observed in Sim1, Sim2, and Sim4, where the adjacent cavity was either not explored or only transiently sampled. In Sim3 and Sim5, dissociation proceeded via the cavity-assisted pathway. Notably, in Sim 5, BCOR remained bound within the adjacent cavity for a significant duration, resulting in delayed dissociation and a distinct 2D FEL.

**Figure 5:**
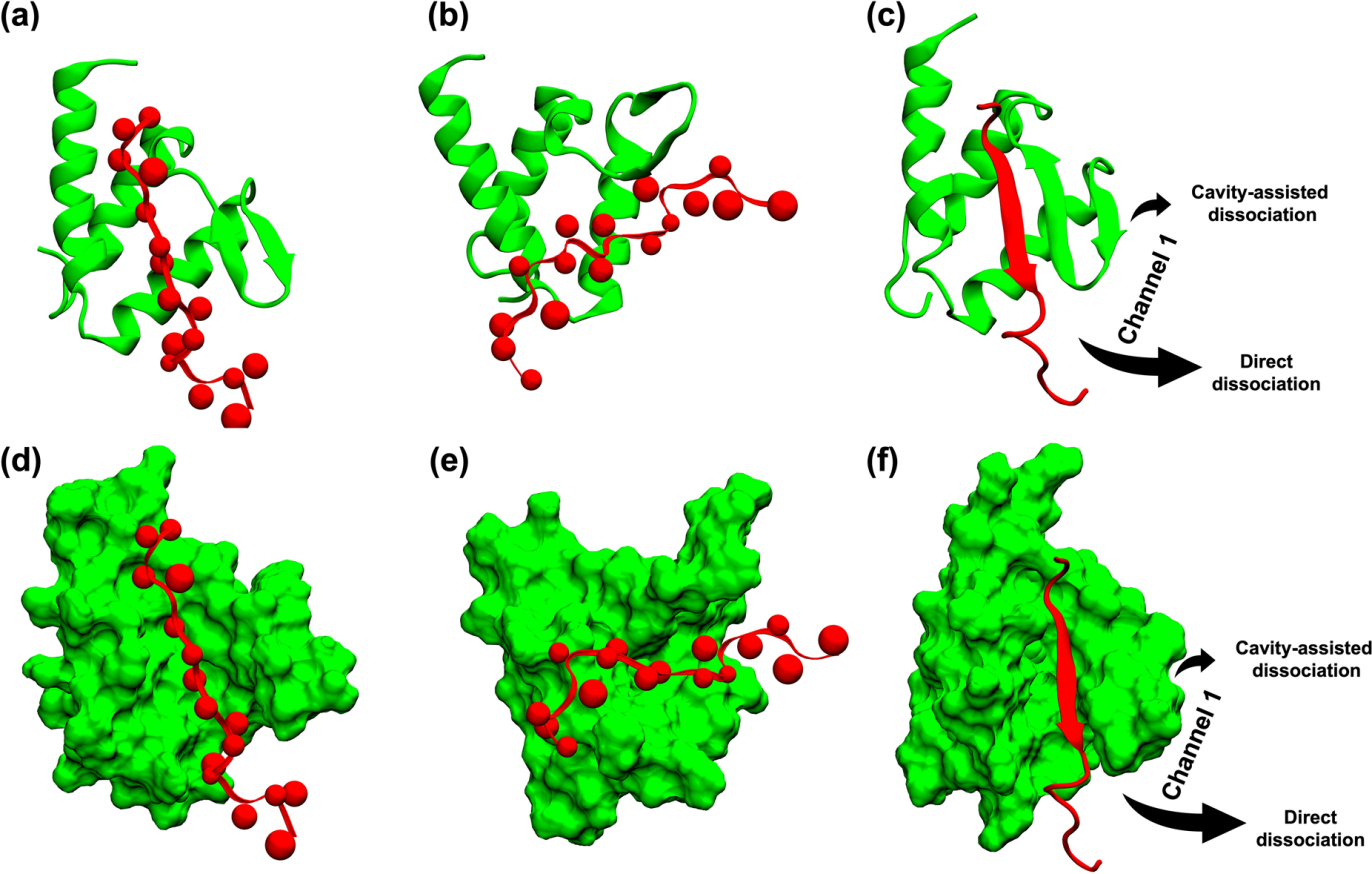
Schematic representation of BCOR dissociation mechanisms: (a, d) direct dissociation, (b, e) cavity-assisted dissociation, and (c, f) combined labeled dissociation pathway through channel 1. AF9 secondary structure is shown as a cartoon and surface (green), while BCOR is represented as beads and ribbons (red). The position of BCOR in the complex indicates its dissociation site.

In the bound state, β2 and α3 form a hydrophobic pocket that accommodates BCOR. The dynamics of β1 and β2 facilitate dissociation by opening the mouth of the binding pocket and making the adjacent cavity accessible to BCOR. The tendency of BCOR to transiently occupy this cavity is facilitated by electrostatic complementarity between its positively charged C-terminus and the negatively charged cavity surface, a feature that warrants further detailed analysis. The two dissociation pathways are summarized in Figure 5. Intermediate I2 in Figure 4c,d illustrates BCOR binding in the alternate cavity before dissociation through channel 1, with hydrophobic interactions stabilizing BCOR within this cavity. Overall, the direct dissociation pathway was observed more frequently and thus can be considered the dominant dissociation route. The direct dissociation of BCOR through channel 1 with time is shown in Figure 3d.

#### 2.2.2 CBX8 Peptide Exhibits Relatively Strong Binding with AF9 and Dissociates via Two Distinct Channels

In five independent 500 ns PPI-GaMD production simulations, the complete dissociation of CBX8 from the binding pocket of AF9 was observed within 400 ns, except for simulation 5. Across the five simulations, the boost potentials were consistent, with average values ranging from ∼8.36 to 9.72 kcal/mol and standard deviations (SDs) between ∼3.23 and 3.81 kcal/mol (Table S8). We then combined all PPI-GaMD production runs to generate free energy profiles for dissociation of CBX8 from AF9 using energetic reweighting (see Section 4).

The COM distance and native contacts between AF9 and CBX8, as well as the peptide RMSD of CBX8 and the distance between the Cα atoms of F543 and I337, were chosen as collective variables to construct the 2D FEL of the AF9–CBX8 complex. These metrics were monitored across all simulations to track peptide dissociation (Figure S7). In Sim2, Sim3, and Sim5, CBX8 RMSD initially increased to ∼10 Å and remained stable until 400 ns, followed by a sudden rise between 400 and 450 ns, indicating dissociation (Figure S7a). In contrast, Sim1 and Sim4 showed an RMSD increase beginning around 100 ns, with dissociation occurring between 250 and 350 ns. The COM distance and interatomic distance plots followed the same trend (Figures 6a and S7b,c). The average number of native contacts between AF9 and CBX8 decreased sharply during the first 50 ns, followed by a gradual decline until ∼310 ns, after which native contacts were nearly absent, consistent with complete peptide dissociation (Figure 6b). The delayed dissociation of CBX8 relative to BCOR suggests stronger binding of CBX8 to AF9, consistent with experimental observations^8^ (Figures 3a and 6a).

**Figure 6:**
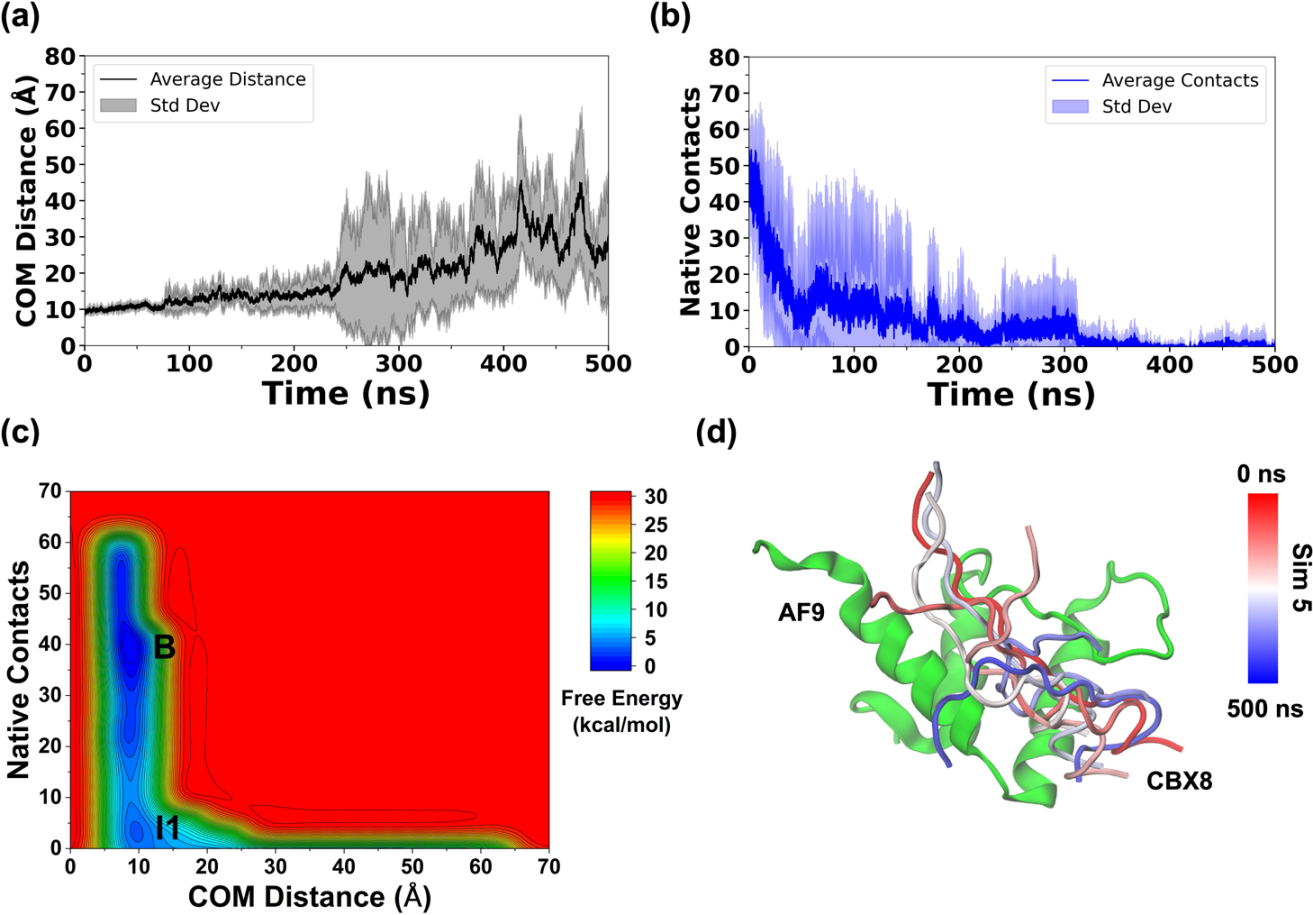
Plots of (a) average COM distance, (b) native contacts between AF9 and CBX8, and (c) combined free-energy landscape of CBX8 dissociation from AF9. The low-energy states are labeled as “Bound” (B) and “Intermediate” (I1), and (d) the dissociation pathway of CBX8 in Simulation 5.

The 2D FELs of individual simulations constructed using COM distance and native contacts revealed well-defined energy minima corresponding to the bound state and intermediate states (Figure S8). The energy minima corresponding to the bound state with native contacts greater than 40 were only observed in Sim 1 and 4. While minima belonging to the bound state with native contacts less than 30 and intermediate states were observed in the FEL of Sim 2–5 (Figure S8). The combined 2D FEL of the AF9–CBX8 complex exhibited a well-defined Bound state “B” centered at a COM distance of 8.6 Å and of 39 native contacts, a lower-energy Intermediate state “I1” at 9.6 Å, and 4 native contacts (Figure 6c).

Additionally, 2D-FELs were prepared by selecting the CBX8 RMSD and F543–I337 distance as reaction coordinates to distinguish additional states. Conformations sampled across all five simulations were highly similar, as indicated by the individual 2D profiles (Figure S9). Broader energy basins for the bound state were observed in Simulations 2, 3, and 5, reflecting longer retention of CBX8 in the bound form (Figure S9b,c,e). The combined 2D FEL of the AF9–CBX8 complex exhibited a stable Bound state “B” centered at RMSD 5.7 Å and distance 5.9 Å, a lower-energy Intermediate state “I2” at 18.03 and 10.05 Å, which is different from that obtained from the FEL constructed using native contacts, and an Unbound state “U” at 49.65 and 47.65 Å (Figure S9f).

DPeak clustering was performed on the concatenated trajectories of the AF9–CBX8 complex, and 81 clusters were obtained (Table S10). A larger number of clusters suggested that multiple conformations of the peptide were sampled in the PPI-GaMD simulations. According to our definition of bound, intermediate, and unbound states, out of 81 clusters, 15 clusters were grouped as bound states, 47 as intermediate, and 19 as unbound states. The centroid structure of each cluster was extracted and mapped with the FEL to identify the bound, intermediate, and unbound states and to identify their molecular interactions. Cluster 0 (C0) was recognized as the Bound conformation, cluster 6 (C6) and cluster 56 (C56) as the Intermediate states (I1, I2). Intermediate I1 has a larger population and represents a major intermediate, while Intermediate I2 has a smaller population and therefore represents a transient state with less probability.

In the Bound state (B), CBX8 retained almost all of the contacts observed in the NMR structure (PDB: 2N4Q). In the intermediate state (I1), some of the native contacts formed between V527-L343, D546-R336, F545–I337, and T541-V339 were preserved, while some new interactions emerged between L514-I337, L504-S332, and L507-A335 (Figure 7a,b). In the Intermediate state (I2), CBX8 lost almost all of the native interactions, including F543–I337, F545–A335, and L457–L333, present in the bound conformation. New interactions were formed between residues H537–L333, T542–I334, T542–A335, L514–R336, and R520–I337 in the intermediate state (Figure 7c,d). Similar to the bound state, the AF9–CBX8 interface is stabilized by a hydrophobic core (L/I/A/P) with additional polar and electrostatic contacts (T and R). Two-dimensional interaction maps highlighting hydrophobic contacts between AF9 and CBX8 in the bound and intermediate states are shown in Figure S10.

**Figure 7:**
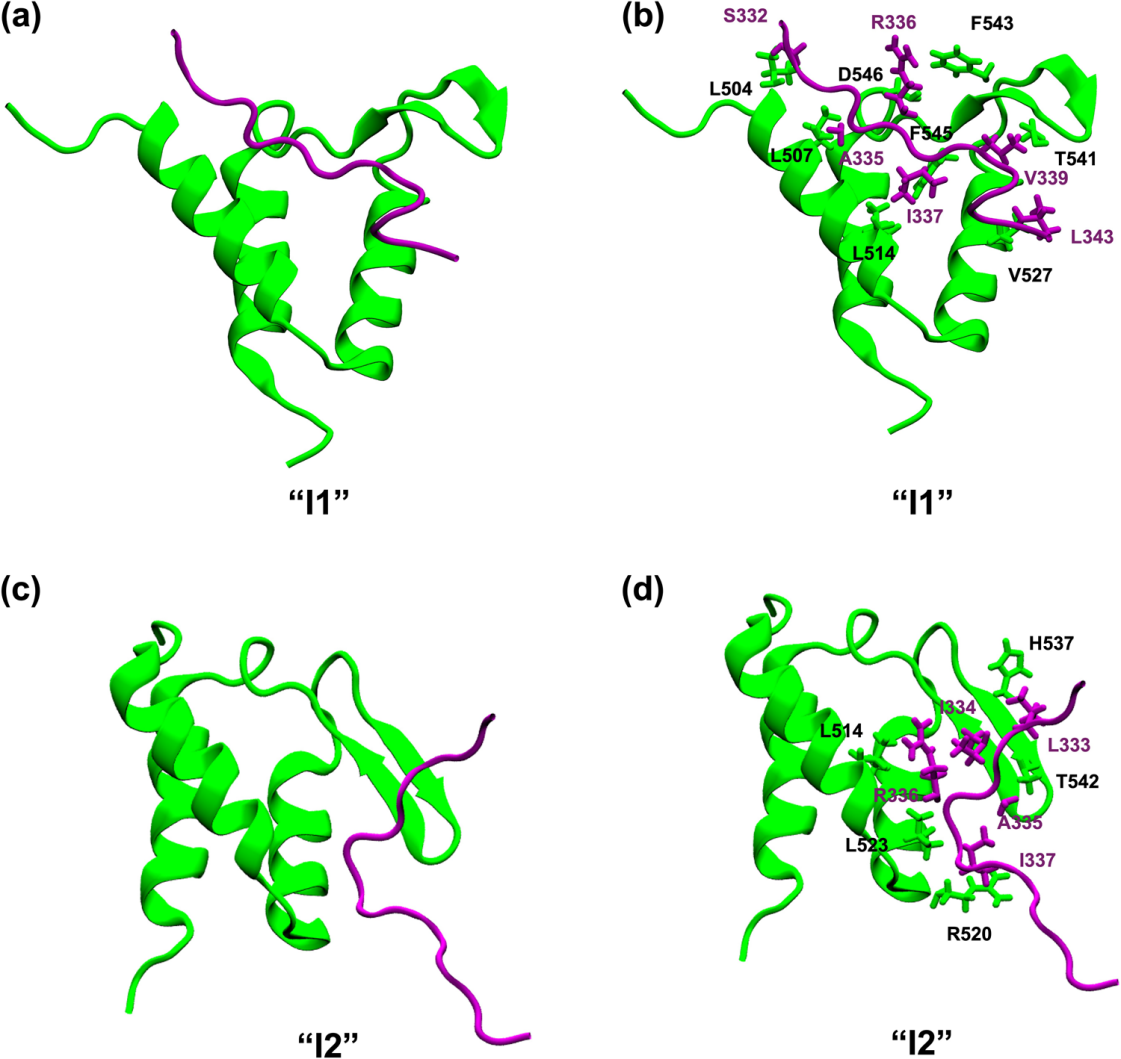
Structure of intermediate states I1 (a), I2 (c), and molecular interactions in intermediate complexes I1 (b), I2 (d) between AF9 and CBX8. The intermediate state was obtained from the DPeak clustering analysis, and after careful mapping onto the free-energy landscape of AF9–CBX8 dissociation.

To elucidate the dissociation pathway of CBX8, the individual trajectories were examined, and two distinct routes were identified. In all cases, dissociation was initiated from the N-terminal region. In Sim1, CBX8 first lost its N-terminal interactions and explored the binding pocket before eventually dissociating through channel 2. In Sim2, CBX8 initially moved away from the pocket and transiently interacted with α-helix 1, while the C-terminal region extended into a nearby cavity, and β-sheets 1 and 2 of AF9 exhibited local fluctuations. CBX8 then gradually exited the pocket, while the β-sheets largely retained their position and secondary structure. In Sim3, dissociation again occurred through channel 2, following brief interactions between the C-terminus of CBX8 and loops generated by partial β-sheet dissolution. By contrast, in Sim4 and Sim5, dissociation proceeded through channel 1. Taken together, these observations indicate that dissociation via channel 1 represents the dominant pathway (Figure 6d), whereas dissociation through channel 2 constitutes a less frequent alternative. The two pathways are illustrated in Figure 8.

**Figure 8:**
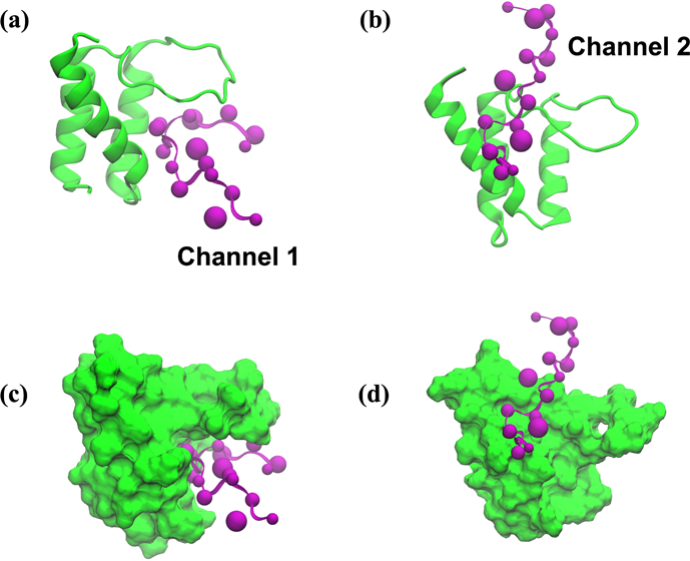
Schematic representation of CBX8 dissociation mechanisms: (a, c) Dissociation via channel 1, and (b, d) Dissociation via channel 2, AF9 secondary structure is shown as a cartoon and surface (green), while CBX8 is represented as beads and ribbon (purple). The position of CBX8 in the complex indicates its dissociation site.

The conformational changes in AF9 were compared in the bound, intermediate, and unbound states (Figure S11), and major fluctuations were observed in the β-hairpin of AF9. In intermediate forms, the β-hairpin was perturbed and moved closer to α-helix 1. In the unbound state, the β-hairpin lost its secondary structure and exhibited severe fluctuations that eventually closed the peptide-binding site of AF9, similar to what was observed in AF9–BCOR complex simulations.

#### 2.2.3 FELs Indicate Strong Binding of DOT1L Peptide to AF9 with Limited Dissociation via Channel 1

Dissociation of DOT1L was assessed based on the peptide RMSD, COM distance, native contacts between AF9 and DOT1L, and the distance between the Cα atoms of residues F543 and I883, as this interaction contributed most significantly to the total binding affinity (Table S6). In Simulations 1–3, DOT1L remained largely bound within the AF9 pocket, showing only modest increases in RMSD, COM distance, and inter-residue distance, indicative of partial conformational rearrangements (Figures 9a and S12a–c). In contrast, complete dissociation was observed in Simulations 4 and 5 between 250 and 350 ns (Figure S12). Collectively, the five GaMD simulations suggest that DOT1L dissociates less readily from AF9 compared with BCOR or CBX8, as full release occurred in only two of the five trajectories. The native contacts between AF9 and DOT1L decreased rapidly during the first 50 ns and then became constant for Simulations 1–3 and completely diminished for Simulations 4–5 (Figures 9b and S12d). The applied boost potentials were comparable across all simulations, with average values of ∼9.04–10.03 kcal/mol and standard deviations of ∼3.08–3.49 kcal/mol (Table S8).

**Figure 9:**
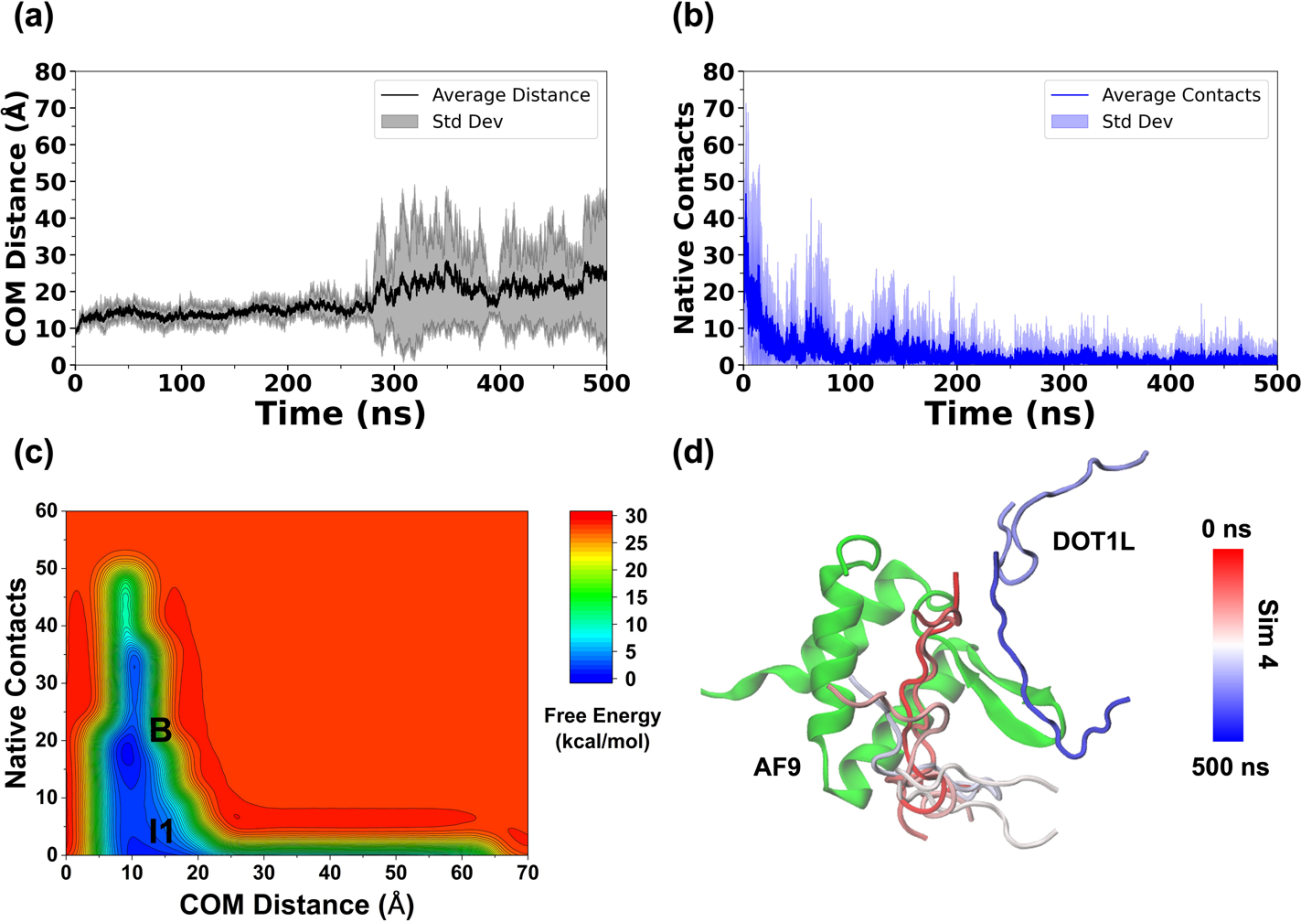
Plots of (a) average COM distance, (b) native contacts between AF9 and DOT1L, and (c) the combined free-energy landscape of DOT1L dissociation from AF9. The low-energy states are labeled as “Bound” (B), and “Intermediate” (I1), and (d) the dissociation pathway of DOT1L in Simulation 4.

2D FELs (both individual and combined) were generated for five AF9–DOT1L PPI-GaMD simulations using COM distance and the native contacts between AF9 and DOT1L as collective variables (Figures 9 and S13). The FELs from Simulations 1, 3, and 4 revealed a broad minimum at lower COM distance, corresponding primarily to bound or partially bound conformations (Figure S13a,c,d). In contrast, Sim2 and Sim4 displayed a minimum at lower native contact values, representing mainly intermediate conformations (Figure S13b, e). The combined 2D FEL of the AF9–DOT1L complex featured a major minimum forming a broad basin centered at a COM distance of 9.5 Å and 17 native contacts (Figure 9c). This broad basin indicated the presence of multiple metastable conformations of comparable energies, reflecting a strong preference for the bound state under the given simulation conditions. Additionally, one lower-energy state was observed, with a minimum centered at (10.6 Å, 1) COM distance and native contact, corresponding to an intermediate state (I1).

Subsequently, 2D-FEL plots were also generated for the AF9-DOT1L complex simulations using DOT1L RMSD and the distance between the Cα atoms of residues F543 and I883 as reaction coordinates (Figure S14). The FELs from Simulations 1 to 3 revealed a broad single minimum at lower RMSD and distance values (Figure S14a–c). In contrast, Sim4 and Sim5 displayed at least two distinct low-energy states representing bound and intermediate conformations (Figure S14d,e). The combined 2D FEL of the AF9–DOT1L complex displayed a dominant minimum forming a broad basin centered at an RMSD of 6.4 Å and an inter-residue distance of 6.1 Å. Additional lower-energy minima corresponding to intermediate state I2 and the unbound state (U) were observed at approximately (29.5 and 25.3 Å) and (44.1 and 42.7 Å), respectively (Figure S14f).

DPeak clustering analysis of the concatenated AF9–DOT1L trajectories identified 88 clusters (Table S11). Upon grouping the total clusters into bound, intermediate, and unbound states, out of 88, 5 clusters belonged to the bound state, 61 clusters to the intermediate state, and 24 clusters to the unbound state. The large number of intermediate conformations observed for CBX8 and DOT1L reflects greater structural diversity and conformational heterogeneity compared with BCOR. Mapping the centroid structures onto the 2D FEL (Figure 9c) revealed that Cluster 9 (C9) represents the bound state, while the most populated Cluster 0 (C0) corresponds to intermediate state I1 (COM distance 10.6 Å, native contacts 1). A minor transient intermediate I2 (Cluster 82) appeared at a higher RMSD and distance but was short-lived (33 frames). Centroid structures of all clusters were analyzed to elucidate the interactions governing DOT1L binding (Figures 10 and S15). Two-dimensional interaction maps between AF9 and DOT1L in bound and intermediate states were generated using MOE (Figure S16) and correlated with the 3D structures. In the bound state (C9), DOT1L’s largely hydrophobic core (L879, P880, V881, I883, L885, A886, S887, V880) was stabilized by complementary hydrophobic residues on AF9 (L514, M515, I538, F543, F545) and several polar/charged residues (H511, Q524, T541, Y503, R520, D544), with F543–I883 forming the most critical contact. S887 formed a persistent hydrogen bond with Q524, particularly in Simulation 2 (Figure S17). In dominant intermediate I1 (C0), DOT1L adopted a more compact conformation to interact with structurally altered AF9 β-sheets, with stabilization primarily via hydrophobic contacts (Figure 10c,d). In I1, key interactions were observed between AF9 and DOT1L residues, including I530–I883, M515–L879, L523–L879, V527–V881, and F545–A886. Additionally, DOT1L exhibited intramolecular hydrophobic interactions involving K878–V888, L879–V888, L879–L890, and K878–S887, highlighting the role of self-association in stabilizing the intermediate state (Figure 10d).

**Figure 10:**
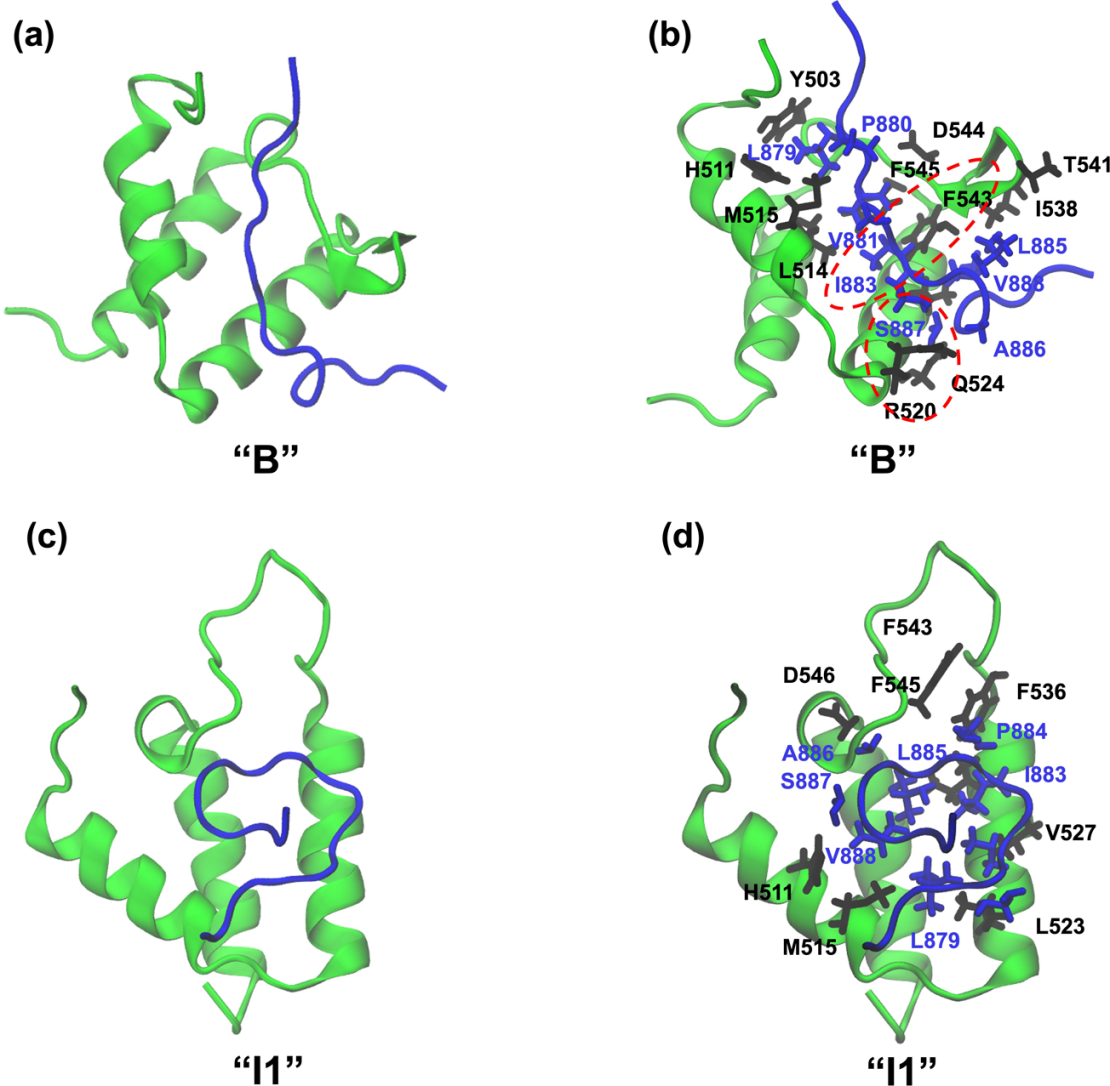
Visualization of structures and molecular interactions of the AF9–DOT1L complex in the bound state (cluster 9) (a,b) and the intermediate state I1 (cluster 0) (c,d) obtained from clustering.

AF9 exhibited significant conformational fluctuations in the dominant intermediate I1, and unbound states compared with the bound state, particularly in the loop following the β-hairpin (Figure 18). Notably, conformational changes in AF9 were more pronounced in the AF9–DOT1L complex than in the AF9–CBX8 or AF9–BCOR complexes, suggesting that DOT1L dissociation requires substantial structural rearrangements of AF9 (Figure S19). The pronounced variability of AF9 across intermediate states underscores its conformational plasticity and highlights the ability of IDRs to adopt multiple conformations to recognize diverse partners. These distinct AF9 conformations observed during the dissociation process reveal potential opportunities for structure-based design of selective inhibitors that stabilize or target specific AF9 conformations, thereby enhancing binding specificity and minimizing off-target effects.

Analysis of the simulation trajectories revealed distinct conformational behaviors of DOT1L across the five GaMD runs. In Simulations 1–3, DOT1L predominantly occupied a partially bound intermediate state. In Sim1, pronounced fluctuations were observed in both the N- and C-termini of DOT1L as well as in the β-sheet region of AF9. During this process, the C-terminal tail of DOT1L folded over its N-terminal segment, while an upward shift of the AF9 loop generated a binding cavity that accommodated the peptide. In Sim2 and Sim3, fluctuations in these regions were less pronounced; AF9 retained its β-sheet architecture, and the C-terminal tail of DOT1L extended into an adjacent cavity. Across these three simulations, DOT1L stabilized in the partially bound state without undergoing complete dissociation. In contrast, Simulations 4 and 5 captured the complete dissociation of DOT1L via channel 1. Dissociation was initiated by cooperative motions of the N- and C-termini, which promoted self-interactions within DOT1L and transiently stabilized intermediates resembling those observed in Sim1 and Sim3. However, these intermediates were short-lived, and the peptide rapidly dissociated. While dissociation via channel 1 is the most frequently sampled pathway for the AF9–DOT1L complex, intermediate states involving intrapeptide self-interactions were consistently observed across simulations. These intermediates may facilitate sampling of alternative dissociation routes, suggesting that AF9–DOT1L dissociation can proceed through multiple parallel pathways rather than through a single deterministic mechanism. Overall, DOT1L displayed a stronger binding affinity toward AF9 compared with that of BCOR and CBX8 under the studied conditions. The termini of DOT1L exhibited pronounced conformational flexibility, whereas its hydrophobic core engaged in both intrapeptide and peptide–AF9 interactions, underpinning its preferential binding to AF9.

For the AF9–DOT1L complex, the FELs display basins broader than those observed for BCOR and CBX8, indicating a more heterogeneous dissociation behavior. Block averaging of the center-of-mass distance across independent PPI-GaMD replicas shows that several trajectories stabilize at similar intermediate separations in the later stages of the simulations, while others reach more dissociated configurations (Table S12). The presence of these stable yet distinct separation regimes across replicas points to multiple metastable states along the dissociation pathway.

Although the free-energy surfaces for AF9–DOT1L are not fully converged, and complete dissociation is not observed in all replicas, the simulations consistently identify reproducible low-energy ensembles and dissociation intermediates. Together with the robustness of the landscapes to reweighting parameters and the identification of structurally coherent clusters within these basins, these observations suggest that the broad DOT1L basins arise from intrinsic landscape ruggedness and pathway-dependent kinetic trapping while still providing robust mechanistic insight into dissociation pathways.

To assess whether enhanced sampling affects peptide–AF9 interactions, we analyzed bound-state ensembles obtained from PPI-GaMD and compared them with the experimental NMR structures (Figure S20). Although partial and transient secondary-structure rearrangements of the peptide were observed, the overall interfacial contact pattern remained stable. The dominant native contacts were preserved in the bound conformation across the three AF9 complexes, indicating that enhanced sampling increases conformational flexibility without qualitatively altering the native binding mode or the interactions stabilizing the complex.

### 2.3 Comparative Analysis of the Dissociation Mechanisms of BCOR, CBX8, and DOT1L

Analysis of the dissociation pathways of BCOR, CBX8, and DOT1L from AF9 revealed pronounced differences in both the timing and mechanism of dissociation. The concatenated 2D FELs of the three complexes exhibited distinct profiles (Figure 11). The FEL of BCOR was characterized by two distinct intermediate states, whereas CBX8 and DOT1L showed comparatively broader landscapes, each containing a single intermediate state. Low-population metastable states were also identified for all three complexes in the FELs defined by peptide RMSD and inter-residue distances. Notably, the BCOR complex sampled unbound conformations more frequently, while CBX8 and DOT1L predominantly remained in bound or partially bound states. The intermediate states in each complex were stabilized through hydrophobic contacts, as shown in 2D interaction plots. The intermediate states observed from clustering analysis were structurally heterogeneous and exhibited varying populations across the different systems. These intermediates correspond to metastable ensembles sampled along multiple dissociation pathways and are not necessarily obligatory checkpoints along the dissociation process. Moreover, highly populated intermediates are associated with dominant pathways, whereas less populated intermediates likely represent alternative dissociation routes.

**Figure 11:**
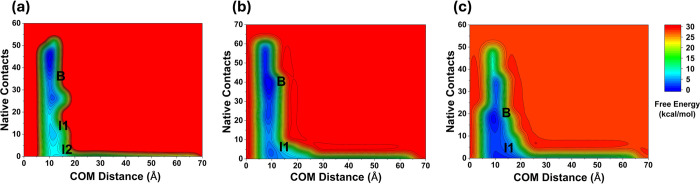
Comparison of combined FEL of (a) AF9–BCOR, (b) AF9–CBX8, and (c) AF9–DOT1L. The low-energy states are labeled as “Bound” (B) and “Intermediate” (I1, I2).

To better understand the origin of these differences, we compared the peptide sequences and their binding modes to those of AF9 (Figure 12). The electrostatic surface representations revealed distinct binding poses that align with the observed hierarchy of binding strengths (BCOR < CBX8 < DOT1L). BCOR features a positively charged C-terminal tail and a discontinuous hydrophobic surface. The presence of K1195 and E1199 interrupts the hydrophobic stretch, orienting BCOR to interact favorably with charged patches on AF9 and allowing it to form an extended β-sheet. However, its positively charged C-terminal lysines (K1206, K1207) preferentially interact with a negatively charged cavity adjacent to the binding pocket, facilitating dissociation through direct or cavity-assisted pathways (Figure 13a). Furthermore, the lack of a continuous hydrophobic interface results in a more open and solvent-exposed binding pocket (Figure 13d).

**Figure 12:**
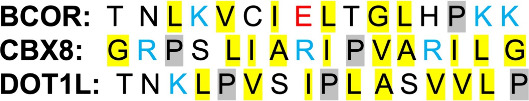
Comparison of the 16 amino-acid sequences of the three AF9-bound peptides. Negatively charged residues are shown in red, positively charged residues in blue, prolines are highlighted in gray, and hydrophobic residues are highlighted in yellow.

**Figure 13:**
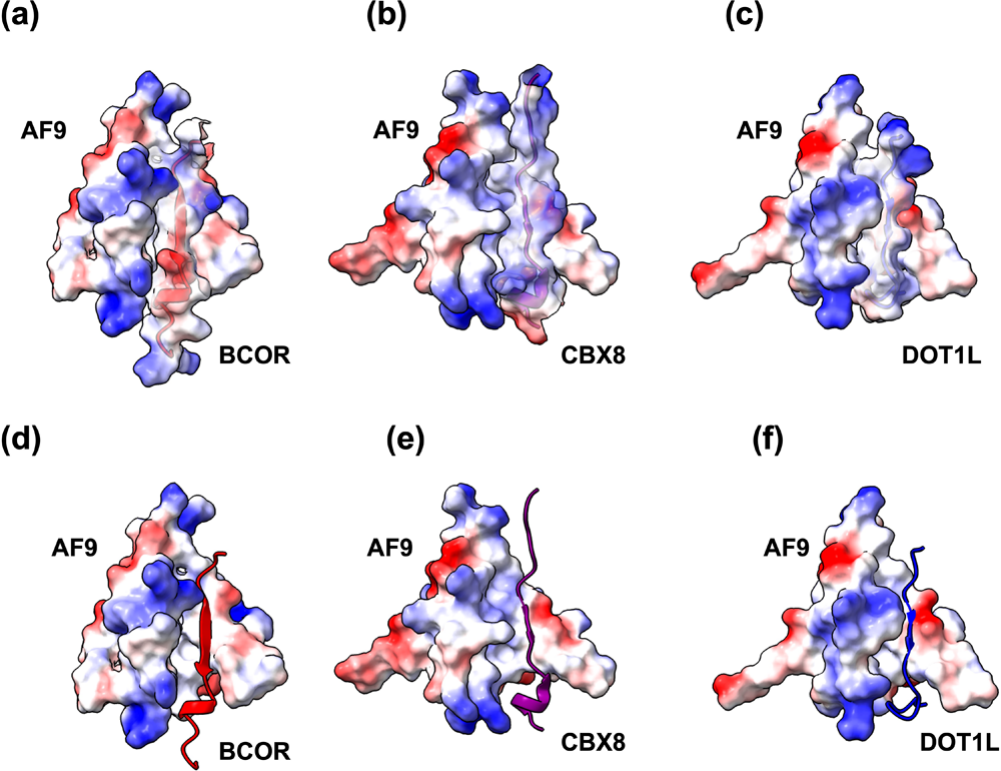
Electrostatic surface representation of the AF9 complex with (a) BCOR, (b) CBX8, and (c) DOT1L. Alternative representation to show the binding pocket in the AF9 complex with (d) BCOR, (e) CBX8, and (f) DOT1L. AF9 is represented as an electrostatic surface while peptides are represented by both cartoons and electrostatic surface.

In contrast, CBX8 and DOT1L present extended hydrophobic surfaces that interact more effectively with AF9’s hydrophobic groove. CBX8 carries multiple arginine residues (R330, R336, and R341), which create electrostatically favorable but partially offset interactions. For example, R336 engages in stabilizing contacts, whereas R330 remains outside the pocket due to repulsion (Figure 13b). Consequently, CBX8 forms a shallow binding pocket stabilized primarily by hydrophobic packing (Figure 13e), allowing dissociation through both channels 1 and 2. DOT1L, however, possesses a strongly hydrophobic tail (V888, V889, L890) that is deeply buried within AF9’s hydrophobic pocket, creating the most compact and stable interface (Figure 13c,f). In addition, DOT1L’s hydrophobic residues not only interact with AF9 but also engage in self-association, stabilizing long-lived metastable conformations observed in several simulations. Dissociation of DOT1L occurred mainly through channel 1, aided by favorable interactions between its slightly positive tail and acidic AF9 residues E531 and E532.

Taken together, these results highlight the dual role of sequence: hydrophobic residues determine the depth and stability of the bound pocket, while electrostatics modulate the preferred dissociation pathway. BCOR, with a discontinuous hydrophobic interface and disruptive C-terminal charges, readily escapes the pocket, explaining its weaker binding. CBX8 achieves intermediate stability by balancing hydrophobic packing with limited electrostatic contributions. DOT1L, dominated by a continuous hydrophobic surface, forms the most stable and buried complex, consistent with its slowest dissociation and strongest binding affinity.

The DPeak clustering analysis further supports the interpretation derived from the FELs by revealing how conformational heterogeneity is distributed across the physically defined states. Clusters were first grouped into bound, intermediate, and unbound ensembles using center-of-mass distance and native contact criteria consistent with the free-energy analysis, enabling direct comparison across AF9–peptide systems (Table S13). Within this framework, multiple structural clusters map onto the same physical state, indicating substantial conformational diversity within the bound and intermediate ensembles. Notably, the dominant source of heterogeneity differs markedly among the three complexes. For AF9–BCOR, only a small number of clusters populated the bound (6 clusters) and intermediate states (9 clusters), reflecting a narrow ensemble of binding conformations. These states lack deep hydrophobic burial and are destabilized by electrostatic interactions, resulting in shallow free-energy minima, from which dissociation occurs readily. In contrast, both AF9–CBX8 (47 clusters) and AF9–DOT1L (61 clusters) exhibited a pronounced expansion of intermediate-state clusters, indicating increased structural flexibility and the presence of multiple structurally distinct, yet physically related, intermediate conformations along the dissociation pathway.

Among these, AF9–DOT1L displayed the largest intermediate ensemble, consistent with a highly rugged binding landscape characterized by alternative hydrophobic packing modes and metastable intermediates. Such landscape ruggedness increases the likelihood of kinetic trapping and prolongs the residence time, thereby strengthening binding. AF9–CBX8 occupied an intermediate regime, with a moderately expanded intermediate ensemble and correspondingly reduced stability relative to DOT1L. While the total number of clusters provides a compact measure of overall conformational heterogeneity, the state-resolved distribution of clusters is more physically informative and directly links the observed landscape features to dissociation behavior. Together, these trends mirror the experimentally observed binding hierarchy: DOT1L > CBX8 > BCOR.

Overall, our results suggest that the interplay of sequence-encoded hydrophobic continuity, charge distribution, and conformational heterogeneity governs both the binding strength and dissociation pathways of AF9’s partner peptides. This mechanistic insight not only rationalizes their relative affinities but also underscores how subtle sequence variations in IDRs can dictate the partner selectivity and binding kinetics.

### 2.4 Druggable Pockets Emerging from AF9 Conformational Plasticity

PPIs have traditionally been regarded as challenging therapeutic targets because of their shallow and extended binding interfaces.^27^ In this context, our analysis revealed that AF9 exhibited pronounced conformational plasticity, leading to the emergence of transient and well-defined binding pockets in intermediate states along the dissociation pathway. By systematically analyzing prominent intermediate conformations sampled along the dissociation pathways of the AF9–BCOR, AF9–CBX8, and AF9–DOT1L complexes, Fpocket identified 9, 6, and 5 pockets, respectively, on the AF9 surface (Figure 14). Characterization of these pockets using geometric and physicochemical descriptors, including druggability score, hydrophobicity, and pocket volume, allowed for the identification of the most promising sites for inhibitor design.

**Figure 14:**
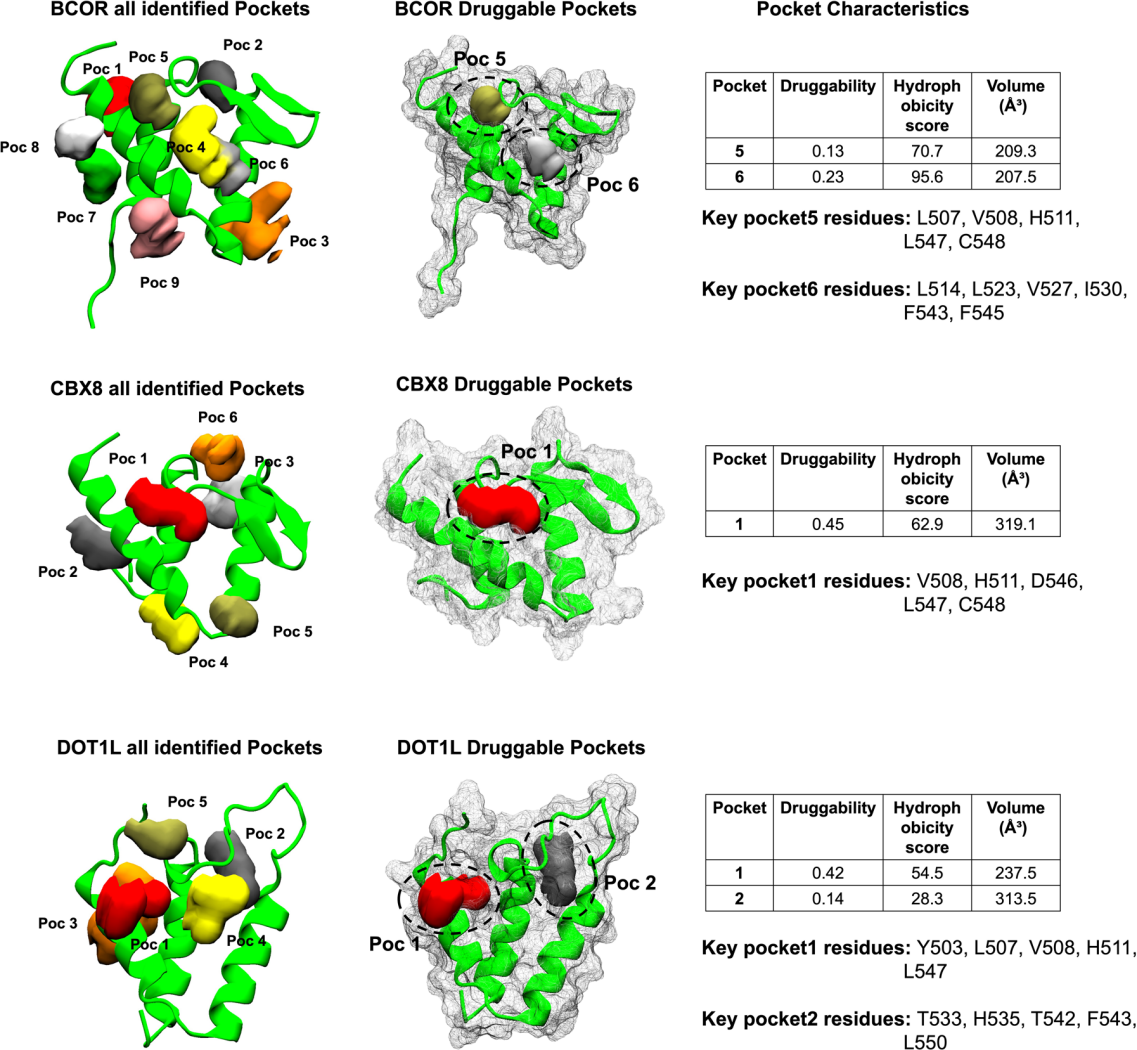
Conformationally induced druggable pockets on the AF9 surface were revealed by intermediate states of the AF9–BCOR, AF9–CBX8, and AF9–DOT1L complexes, together with druggability metrics and annotation of key pocket-forming residues relevant for inhibitor design.

In the AF9–BCOR intermediate conformations, two pockets (pockets 5 and 6) exhibited favorable druggability scores and were located at the interface between the α-helices and β-sheets. Pocket 5 is primarily formed by residues L507, V508, H511, L547, and C548, while pocket 6 involves residues L514, L523, V527, I530, F543, and F545. Both pockets have volumes of approximately 210 Å^3^, comparable to those of established small-molecule binding sites, and are characterized by pronounced hydrophobicity consistent with AF9 partner recognition. Although the absolute druggability scores are modest, as expected for transient and partially open PPI pockets, the combination of favorable pocket volume and strong hydrophobic enclosure suggests that these sites may be amenable to small-molecule binding in intermediate conformations. Notably, pocket 6 exhibits higher hydrophobicity and druggability among the two, highlighting it as the most promising candidate site in the AF9–BCOR complex.

In the AF9–CBX8 intermediate state, a single highly druggable pocket (pocket 1) was detected, lined by residues V508, H511, D546, L547, and C548. The identified pocket (pocket 1) was characterized with a druggability score of 0.45 and a volume of approximately 319 Å^3^, placing it among the most favorable sites detected across all complexes. This pocket displayed a balanced hydrophobic character and sufficient enclosure to accommodate drug-like ligands. Notably, both the pocket size and the druggability score exceed those observed for the AF9–BCOR pockets, highlighting this site as a particularly promising candidate for small-molecule targeting in the AF9–CBX8 complex.

For the AF9–DOT1L complex, two druggable pockets (pockets 1 and 2) emerged from substantial structural fluctuations in the β-sheet region, with pocket 1 defined by residues Y503, L507, V508, H511, and L547 and pocket 2 by residues T533, H535, T542, F543, and L550. Notably, many of the key lining residues originate from flexible segments that underwent substantial reorganization during partner dissociation, generating mixed hydrophobic and polar environments conducive to ligand anchoring. The predominance of hydrophobic residues within these pockets is consistent with the central role of hydrophobic interactions in AF9 partner binding. Importantly, each AF9 conformation examined contains at least one pocket with distinct residue composition and geometry, indicating the presence of conformation-specific sites that could, in principle, be exploited for partner-selective modulation.

Collectively, these observations establish a structural connection between AF9 conformational dynamics and the emergence of potentially druggable pockets. While experimental validation will be required to assess the accessibility and targetability of these sites, our results suggest that intermediate conformations may provide additional opportunities for targeting AF9 beyond the native binding interface. More generally, this analysis highlights the value of exploring intermediate-state pocket formation when investigating dynamic PPI systems.

## 3 Conclusion

In this study, all-atom PPI-GaMD simulations were applied to elucidate the atomistic mechanism of peptide partner dissociation in three AF9 PPI complexes linked to MLL-rearranged (MLL-r) leukemia. We successfully captured the spontaneous dissociation of all three peptide partners—BCOR, CBX8, and DOT1L—from AF9, revealing distinct dissociation pathways whose relative stabilities align well with the experimental observations.^8^ MM-GBSA analysis, together with the PPI-GaMD results, indicated that BCOR binds most weakly to AF9, CBX8 shows intermediate affinity, and DOT1L exhibits the strongest binding. Per-residue decomposition analysis further revealed that van der Waals interactions dominate the stabilization of AF9–partner complexes, with AF9 residues F543 and F545 contributing the most favorably to the overall binding free energy.

Our PPI-GaMD simulations revealed that sequence-encoded differences among BCOR, CBX8, and DOT1L give rise to distinct 2D FELs and dissociation channels. Peptide dissociation proceeded through two main routes, involving metastable intermediate states stabilized by transient hydrophobic contacts. Notably, residues forming these contacts, such as V881 and I883 in DOT1L, have been experimentally shown to be critical for AF9 binding.^9^ These results suggest that hydrophobic contacts transiently exposed in intermediate states represent mechanistic features that shape AF9 partner recognition and dissociation. The intrinsically disordered binding region of AF9 exhibits pronounced conformational adaptability, particularly in complex with DOT1L, highlighting its capacity to remodel during partner disengagement. Our pocket analysis further revealed that this structural plasticity gives rise to conformation-dependent binding pockets with distinct geometric and physicochemical features. The proposed binding pockets may represent candidate sites for the future design of small-molecule inhibitors, although their ligand-binding potential and selectivity will require validation through dedicated binding simulations and experimental studies. The conformational flexibility of AF9 observed in PPI-GaMD simulations is consistent with previous 200 ns REMD simulations of the apo protein,^12^ which showed total loss of β-sheet secondary structure in the binding region, indicating inherent structural plasticity. Circular Dichroism experiments further support these findings, showing minimal β-content in the AF9-AHD region.^10^ The agreement between PPI-GaMD, REMD, and experimental data indicates that the structural remodeling observed is an intrinsic property of AF9 rather than an artifact of enhanced sampling.

Comparative analysis of the peptide sequences and binding poses revealed that intrinsic sequence features govern the AF9-binding preferences. BCOR, with its discontinuous hydrophobic surface and negatively charged C-terminus, forms fewer stable contacts and readily dissociates, consistent with its weak affinity. Our previous study also underscored the critical role of BCOR’s C-terminal region in forming the AF9–BCOR complex through the construction of its BFEL.^12^ CBX8 exhibits intermediate binding stability by balancing hydrophobic packing with modest electrostatic interactions. In contrast, DOT1L’s continuous hydrophobic surface forms a deeply buried and kinetically rugged interface, stabilizing multiple metastable states and leading to its strongest binding. Collectively, these results demonstrate that the interplay among hydrophobic continuity, charge distribution, and conformational heterogeneity governs both binding affinity and dissociation kinetics. This conclusion is further supported by quantitative energetic and surface-area analyses, which showed that progressively greater nonpolar burial and hydrophobic stabilization across the complexes directly track the binding hierarchy, with DOT1L exhibiting the strongest hydrophobic contribution.

Beyond the three peptide partners examined here, AF9 engages additional binding partners, including members of the AF4 family, through its AHD. Notably, DOT1L and AF4 peptides share a closely related hydrophobic recognition motif (LxVxIxLxxL/V) and bind the AF9 AHD with comparable affinities, consistent with a conserved mode of molecular recognition. In light of this sequence and affinity similarity, we propose that the dissociation mechanism characterized for the DOT1L complex is likely representative of AF4–AF9 unbinding as well. More broadly, our results suggest that the dissociation framework identified in this study is applicable to a wider class of AF9 partners that engage the AHD through analogous hydrophobic motifs, with partner-specific sequence variations primarily modulating dissociation kinetics and the relative stability of intermediate states. Although the simulations presented here were designed to probe dissociation rather than spontaneous association, the unbinding trajectories provide indirect insights into the association process. Dissociation consistently proceeded through a small number of preferred pathways, indicating an anisotropic binding interface with well-defined interaction channels. The persistence of conserved native contacts in dominant intermediate states suggests that these residues are likely to serve as initial recognition or anchoring sites during association. In addition, the substantial conformational rearrangement of AF9 upon peptide release indicates that the domain is not preorganized for binding, supporting an induced-fit or conformational selection mechanism.

The PPI-GaMD simulations were initiated from a representative structure (Model 1) of the NMR ensemble, selected because it satisfies all experimental restraints and captures the dominant bound-state geometry. The peptide segments retained in the truncated constructs exhibit limited structural variability across the NMR ensemble, supporting the use of this model as a suitable starting conformation. We note, however, that initiating simulations from a single NMR conformer may, in principle, bias sampling toward a specific region of conformational space. In the present study, this potential bias is mitigated by the use of PPI-GaMD enhanced sampling and multiple independent replicas. Importantly, dissociation pathways and intermediate states were reproducible across replicas, indicating that the qualitative features of the dissociation mechanism are not artifacts of the initial model selection.

The use of truncated 16-residue peptides represents a reduced model of the full-length binding partners and may not fully capture absolute binding energetics or dissociation kinetics under physiological conditions. Nevertheless, because these peptides encompass the experimentally validated AF9-binding core, they provide a robust framework for dissecting conserved interfacial interactions and relative dissociation pathways and mechanistic features. A consistent agreement between our results and experimental data also validates the chosen system representation. Extension to full-length peptides will be important for quantitatively assessing kinetic and thermodynamic effects. Accurate estimation of absolute binding free energies and kinetic rate constants for BCOR, CBX8, and DOT1L would require capturing multiple association and dissociation events, which will be the focus of future work.

In summary, this study provides the first atomistic description of AF9–partner dissociation, revealing how sequence-encoded features dictate the stability and kinetics of the BCOR, CBX8, and DOT1L interactions. These mechanistic insights establish a rational computational framework for the design of selective peptide or covalent inhibitors^28,29^ targeting AF9-mediated interactions—offering new opportunities to therapeutically modulate AF9-driven transcriptional complexes in MLL-rearranged leukemia.

## 4 Methods

### 4.1 Preparation of Starting Structures

The starting structures of the AF9 complex with minimally interacting regions of BCOR, CBX8, and DOT1L were obtained from the RCSB PDB IDs: 6B7G,^8^ 2N4Q,^8^ and 2MV7,^9^ respectively. From the ensemble of 10 NMR structures of each complex, Model 1 was selected for simulations, as it was designated in the PDB entry as the best representative conformer of the NMR ensemble. The peptides BCOR, CBX8, and DOT1L have different chain lengths with 33, 24, and 25 residues, respectively. To compare their dissociation timelines and to reduce the complexity in PPI-GaMD simulations, the peptide length was kept at 16 residues. The peptides were trimmed by removing the residues that showed very high fluctuations in the 10 NMR models of each complex. In BCOR, N-terminal residues 1175–1191 (17 residues); in CBX8, N-terminal residues 326–328 and C-terminal residues 345–349 (8 residues in total); and in DOT1L, C-terminal residues 892–900 (9 residues) were truncated to ensure equal peptide lengths across all constructs.

### 4.2 Classical Molecular Dynamics Simulations

The classical molecular dynamics (CMD) simulations were performed on NMR structures of AF9–BCOR,^8^ AF9–CBX8,^8^ and AF9–DOT1L^9^ complexes, using GROMACS 2023.^30^ CHARMM36m force field^31,32^ was used for obtaining the protein parameters. The CHARMM36m force field effectively reproduces the conformational properties of intrinsically disordered proteins (IDPs), capturing both structured and flexible regions with high accuracy.^33,34^ All the AF9 complexes were placed at the center of cubic boxes, and the distance between protein atoms and the box edge was kept at 1 nm to avoid any interaction with their periodic image during simulation. The complexes were solvated in TIP3P^35^ water molecules and were neutralized by adding 0.15 M sodium chloride. Energy minimization was performed on the complexes using the Steepest Descent method to remove any bad contacts. First, the equilibration was carried out in the NVT ensemble to maintain a constant temperature of 303.15 K for 500 ps, followed by equilibration under the NPT ensemble to maintain a constant pressure of 1 bar for 20 ns. After monitoring the convergence of temperature, pressure, density, etc., the complexes were simulated for 200 ns under the NPT ensemble. A 2 fs time step was used for the simulations. Temperature was controlled using the v-rescale thermostat,^36^ while pressure coupling was maintained with the c-rescale method.^37^ Long-range electrostatic interactions were treated using the Particle Mesh Ewald (PME) approach,^38^ with a cutoff distance of 1.2 nm applied to both electrostatic and van der Waals interactions. Bond constraints involving hydrogen atoms were handled using the LINCS algorithm,^39^ and periodic boundary conditions were imposed in all three spatial dimensions.

### 4.3 PPI-GaMD Simulations

All the PPI-GaMD simulations^22^ on AF9–BCOR, AF9–CBX8, and AF9–DOT1L complexes were performed using AMBER22 ^40^ with the trimmed peptide complexes prepared as described previously. In PPI-GaMD simulations, first CMD simulations were performed, followed by equilibration and production under PPI-GaMD settings. The simulation files for classical MD were generated using CHARMM-GUI,^41^ and the protein–peptide complexes were defined using the CHARMM36m force field.^31^ The N- and C-termini of peptides (BCOR/CBX8/DOT1L) were kept charged (NH3^+^ and COO^–^), and the N and C-termini of AF9 were capped with neutral residues (acetyl and methyl amide). The peptide termini were kept in charged states to reflect physiological conditions and avoid artificial stabilization of the bound complex, thereby promoting realistic solvation and conformational flexibility important for dissociation sampling in enhanced simulations. This choice is supported by a recent Pep-GaMD study by Jinan et al.,^42^ who showed that zwitterionic peptides more reliably reproduce experimental structures across multiple peptide–protein systems. While altering terminal charges may influence dissociation pathways, PPI-GaMD simulations typically retain charged termini, and this model is considered most physically representative for small biological peptides. The complexes were placed in a cubic box filled with TIP3P water molecules, which extended for 18 Å from the complex surface. The systems were neutralized by adding 0.15 M sodium chloride. The schematic of the AF9–CBX8 complex in the solvated box is shown in Figure S21. The systems were energy minimized for 5000 steps to remove any bad contacts, followed by two-step equilibrations; first under the NVT ensemble for 125 ps at 310 K and then under the NPT ensemble to maintain constant pressure at 1 atm. Before running PPI-GaMD simulations, a cMD simulation was performed on each system for 20 ns at a temperature of 310 K and a pressure of 1 bar. For temperature and pressure control, the Langevin thermostat^43^ and Berendsen barostat^44^ were used, respectively. The PME method was used to compute the long-range electrostatic interactions, and a cutoff of 9 Å was chosen for calculating the short-range electrostatic and van der Waals interactions.^38^ After cMD, PPI-GaMD equilibration was performed on each complex system for 51 ns. To observe the dissociation of peptides during PPI-GaMD production while keeping the boost potential as low as possible for accurate energetic reweighting, the σ_OP_ and σ_OD_ parameters were set to 1.2 and 6.0 kcal/mol, respectively, for all three AF9 complexes. A total of five PPI-GaMD simulations of 500 ns were run per system to ensure the reliability and reproducibility of the results. The trajectories were saved every 0.2 ps for analysis. The PPI-GaMD simulations on the three AF9 complexes are summarized in Table S8.

### 4.4 Simulation Analysis

The binding free energies of the three AF9 complexes were calculated with the MM-GBSA method^24^ (see Supplementary methods). The buried nonpolar surface area associated with hydrophobic residues (A, V, L, I, M, F, W, P, and C) was calculated using the GROMACS SASA tool. The nonpolar solvent-accessible surface area was evaluated separately for AF9, the peptide, and the AF9–peptide complex, and the buried nonpolar surface area was then obtained according to eq 1.

$$\mathrm{Buried}\;\mathrm{Non}‐\mathrm{polar}\;\mathrm{Surfae}\;\mathrm{Area}=(\mathrm{Non}‐\mathrm{polar}\;\mathrm{Surface}\;\mathrm{area}{)}_{\mathrm{AF}9}+(\mathrm{Non}‐\mathrm{polar}\;\mathrm{Surface}\;\mathrm{area}{)}_{\mathrm{Peptide}}-(\mathrm{Non}‐\mathrm{polar}\;\mathrm{Surface}\;\mathrm{area}{)}_{\mathrm{Complex}}$$ 1

For monitoring the dissociation of peptides from AF9, simulation analyses were performed using CPPTRAJ^45^ and VMD.^46^ The peptide RMSD, COM distance between AF9 and the peptide, distance between the Cα atoms of residues forming the strongest contact in the NMR structure, and native contacts between AF9 and the peptide were chosen as reaction coordinates for the FEL. Native contacts between AF9 and the peptide were computed using CPPTRAJ with respect to the reference bound structure. A native contact was defined when any heavy atom from a peptide residue was within 4.5 Å of any heavy atom from an AF9 residue. Molecular operating environment (MOE)^26^ was used to identify the residue pairs of AF9 and the peptide that contribute significantly to the binding affinity, and the pair contributing most to the binding was selected for the distance calculation. The 2D FELs were reweighted using the PyReweighting toolkit^47^ (See Supplementary Methods). A bin size of 4 Å was used for peptide RMSD, native contacts, COM distance, and Cα atom distances. The reweighting was done by setting a cutoff of 500 frames in one bin.

The clustering on the concatenated trajectories of each AF9–peptide complexes was performed using the Density Peak^25^ (DPeak) clustering algorithm with a sieve value of 20 and an epsilon parameter of 2 in CPPTRAJ to identify the low-energy states in the 2D free energy profiles. These parameters were optimized using the AF9–BCOR system and were selected to balance adequate conformational sampling with computational efficiency while minimizing the generation of low-population clusters that are unlikely to be biologically meaningful. The identified low-energy states were carefully mapped as bound, intermediate, and unbound states, and the interactions between AF9 and peptides were visualized using VMD,^46^ ChimeraX,^48^ and MOE.^26^ The FEL plots were prepared using OriginPro2024.^49^ Intermediate states were defined as configurations retaining one-fourth or fewer of the native contacts relative to the NMR structure, while maintaining a center-of-mass or inter-residue distance of less than 20 Å.

Convergence of key observables was assessed by using block averaging. PPI-GaMD trajectories were divided into four equal time blocks, and the mean and standard deviation of the center-of-mass distance was calculated within each block. Stabilization of block-averaged values in the later portions of the simulations were used as an indicator of internal convergence and reproducibility across independent replicas.

Druggable binding pockets on the surface of AF9 were identified using Fpocket,^50^ and pockets with high druggability scores were selected as potential targets for inhibitor design.

## Supplementary Material



## Data Availability

The free software tools used in this study, including CHARMM-GUI (https://www.charmm-gui.org), UCSF ChimeraX (https://www.cgl.ucsf.edu/chimerax/), Fpocket (https://github.com/Discngine/fpocket), and OriginPro2024 trial version (https://www.originlab.com), are freely available at their websites. The gmx_MMPBSA (https://valdes-tresanco-ms.github.io/gmx_MMPBSA/dev/installation/) is available to use with GROMACS 2023. AMBER2022 for simulations. All the input files required to reproduce the results and the initial as well as final structures are available for free at GitHub (https://github.com/SahaLabGitHub/AF9_BCOR_PPIGAMD).

## Notes

Method S1. PPI-GaMD method, Method S2. Energetic reweighting, Method S3. MM-GBSA method, Table S1. Binding free energies of AF9-peptide complexes, Table S2. Per-residue contribution in AF9-BCOR complex, Table S3. Per-residue contribution in AF9-CBX8 complex, Table S4. Per-residue contribution in AF9-DOT1L complex, Table S5. AF9-BCOR interactions, Table S6. AF9-CBX8 interactions, Table S7. AF9-DOT1L interactions, Table S8. PPI-GaMD simulation details of AF9-peptide complexes, Table S9. DPeak clustering on AF9-BCOR simulation, Table S10. DPeak clustering on AF9-CBX8 simulation, Table S11. DPeak clustering on AF9-DOT1L simulation, Table S12. Block-averaged COM distances for AF9–DOT1L, Table S13. Classification of clusters in different states, Figure S1. Structural properties for replicate simulations of AF9-BCOR complex, Figure S2. 2D-FEL (COM, native contacts) for AF9-BCOR replicate simulations, Figure S3. 2D-FEL (RMSD, F545–V1196 distance) for AF9-BCOR replicate simulations, Figure S4. 2D interaction plots of AF9-BCOR bound and intermediate states, Figure S5. Comparison of AF9 structure obtained from the different states of AF9-BCOR complex, Figure S6. Peptide dissociation pathways of AF9, Figure S7. Structural properties for replicate simulations of AF9-CBX8 complex, Figure S8. 2D-FEL (COM, native contacts) for AF9-CBX8 replicate simulations, Figure S9. 2D-FEL (RMSD, F543–I337 distance) for AF9-CBX8 replicate simulations, Figure S10. 2D interaction plots of AF9-CBX8 bound and intermediate states, Figure S11. Comparison of AF9 structure obtained from the different states of AF9-CBX8 complex, Figure S12. Structural properties for replicate simulations of AF9-DOT1L complex, Figure S13. 2D-FEL (COM, native contacts) for AF9-DOT1L replicate simulations, Figure S14. 2D-FEL (RMSD, F543–I883 distance) for AF9-DOT1L replicate simulations, Figure S15. Intermediate structure (I2) of AF9-DOT1L complex, Figure S16. 2D interaction plots of AF9-DOT1L bound and intermediate states, Figure S17. H-bond formation between Q524-S887 in AF9-DOT1L complex, Figure S18. Comparison of AF9 structure obtained from the different states of AF9-BCOR complex, Figure S19. Comparison of intermediate structures of AF9 in AF9-BCOR/CBX8/DOT1L complexes, Figure S20. Comparison of key interactions in the experimental and PPI-GaMD bound states, and Figure S21. Simulated water box (PDF)
