# Supplementary material for: Mechanism of AF9 (MLLT3)–Partner Dissociation in Mixed-Lineage Leukemia-Rearranged Leukemia
Source: JACS Au. 2026 Mar 7;6(4):2226–43. doi: 10.1021/jacsau.5c01454 (PMC13126186; doi:10.1021/jacsau.5c01454)
Supplement: Supplementary file 1 [file au5c01454_si_001.pdf]

Mechanism of AF9 (MLLT3)–Partner Dissociation in MLL-Rearranged Leukemia

Shilpa Sharma[a], Keya Joshi[b,c], and Arjun Saha[a,*]

[a] Department of Chemistry and Biochemistry, University of Wisconsin-Milwaukee, Milwaukee, Wisconsin 53211, USA

[b] Computational Medicine and Pharmacology, University of North Carolina, Chapel Hill, North Carolina, 27599, USA

[c] Department of Computational Biology and Bioinformatics, University of North Carolina, Chapel Hill, North Carolina, 27599, USA

*Corresponding Email: saha6@uwm.edu

Supplementary Methods

S1. Protein-Protein Interaction-Gaussian accelerated Molecular Dynamics (PPI-GaMD)

Building upon GaMD methodology, a new method called PPI-GaMD[1] is developed for improved sampling of protein-protein interactions. Considering a system of protein L binding to another protein P present in a biological environment E. A total of N atoms is present in the system with their coordinates defined as $r \equiv \{\vec{r}_1, \cdots, \vec{r}_N\}$, and their momenta defined as $p \equiv \{\vec{p}_1, \cdots, \vec{p}_N\}$. The Hamiltonian of the system can be calculated as:

$$H(r,p) = K(p) + V(r), \tag{1}$$

where $K(p)$ denotes the kinetic energy while $V(r)$ denotes the total potential energy of the system, respectively. The potential energy term $V(r)$ is further divided into the following terms:

$$\begin{aligned} V(r) &= V_{P,b}(r_P) + V_{L,b}(r_L) + V_{E,b}(r_E) \\ &+ V_{PP,nb}(r_P) + V_{LL,nb}(r_L) + V_{EE,nb}(r_E) \\ &+ V_{PL,nb}(r_{PL}) + V_{PE,nb}(r_{PE}) + V_{LE,nb}(r_{LE}) \end{aligned} \tag{2}$$

where the bonded potential energies in the protein *P,* protein *L* and environment *E* are denoted as $V_{P,b}$, $V_{L,b}$ and $V_{E,b}$, respectively. The self-non bonded potential energies in protein *P*, protein *L*, and environment *E* are denoted as $V_{PP,nb}$, $V_{LL,nb}$ and $V_{EE,nb}$, respectively. *P-L*, *P-E*, and *L-E's* related non-bonded interaction energies are given as $V_{PL,nb}$, $V_{PE,nb}$ and $V_{LE,nb}$, respectively.

Based on classical molecular mechanics force fields[2,3], the non-bonded potential energies of the proteins *P, L* and environment *E* are calculated as:

$$V_{nb} = V_{elec} + V_{vdW}, \tag{3}$$

where V_{elec} represents the system's electrostatic potential energy while V_{vdW} denotes the system's van der Waals potential energy.

The interaction energy between the protein binding partners is given as $V_{PL,nb}(r_{PL})$. In PPI-GaMD, a boost potential is selectively added to the protein binding partners interaction energy based on the GaMD methodology[4,5,6]:

$$\Delta V_{PL,nb}(r) = \begin{cases} \frac{1}{2} k_{PL,nb} \left(E_{PL,nb} - V_{PL,nb}(r_{PL})\right)^2, & V_{PL,nb}(r_{PL}) < E_{PL,nb} \\ 0, & V_{PL,nb}(r_{PL}) \geq E_{PL,nb} \end{cases} \tag{4}$$

where $E_{PL,nb}$ denotes the threshold for applying the boost potential and $k_{PL,nb}$ represents the harmonic constant. All the other parameters in PPI-GaMD are derived in the same manner as in the previous GaMD method[4-6]. When *E* is set to the lower bound as the system maximum potential energy (E=V_{max}), the effective harmonic force constant k_0 is given as:

$$k_0 = \min(1.0, k_0') = \min\left(1.0, \frac{\sigma_0}{\sigma_V} \frac{V_{max} - V_{min}}{V_{max} - V_{avg}}\right), \tag{5}$$

where V_{max}, V_{min}, V_{avg} and σ_V represents the maximum, minimum, average, and standard deviation of the boosted system potential energy, and σ_0 denotes the user-specified upper limit of

the standard deviation of ΔV to perform accurate reweighting[7]. The harmonic constant is given as $k = k_0 \cdot \frac{1}{V_{max}-V_{min}}$ with the following condition: $0 < k_0 \leq 1$.

Alternatively, when the threshold energy E is set to its upper bound $E = V_{min} + \frac{1}{k}$, k_0 is given as:

$$k_0 = k_0^{''} \equiv (1 - \frac{\sigma_0}{\sigma_V}) \frac{V_{max}-V_{min}}{V_{avg}-V_{min}} \tag{6}$$

given that $k_0^{''}$ is found to be between *0* and *1*. Otherwise, k_0 is calculated using Eqn. (5).

In PPI-GaMD, a second boost potential is also applied on the remaining potential energy of the entire system to increase conformational sampling and facilitate protein rebinding. The second boost potential is calculated using the total system potential energy which excludes the interaction potential between the proteins as:

$$\Delta V_D(r) = \begin{cases} \frac{1}{2} k_D \left(E_D - V_D(r)\right)^2, & V_D(r) < E_D \\ 0, & V_D(r) \geq E_D \end{cases} \tag{7}$$

where V_D denotes the total system potential energy between the protein binding partners, E_D corresponds to the threshold energy for applying the second boost potential and k_D is the harmonic force constant, respectively. This provides us with the dual-boost PPI-GaMD with the total boost potential $\Delta V(r) = \Delta V_{PL,nb}(r_{PL}) + \Delta V_D(r)$.

S2. Energetic reweighting of PPI-GaMD for free energy calculations

To perform energetic reweighting of PPI-GaMD simulations to recover the original free energy landscape, the probability distribution defined along a selected reaction coordinate can be calculated as $p^*(A)$. Given the boost potential $\Delta V(r)$ in each frame of PPI-GaMD simulations, $p^*(A)$ can be reweighted to recover the canonical ensemble distribution, $p(A)$, given as:

$$p(A_j) = p^*(A_j) \frac{\langle e^{\beta \Delta V(\bar{r})} \rangle_j}{\sum_{i=1}^{M} \langle p^*(A_i) e^{\beta \Delta V(\bar{r})} \rangle_i}, \; j = 1, \ldots, M \tag{8}$$

where M represents the number of bins in the simulation frames, $\beta = k_B T$ and $\langle e^{\beta \Delta V(\bar{r})} \rangle_j$ denotes the ensemble-averaged Boltzmann factor of $\Delta V(\bar{r})$ for the simulation frames found in the jth bin. Cumulant expansion to the second order is applied to the ensemble-averaged reweighting factor to reduce the energetic noise[4, 7]:

$$\langle e^{\beta \Delta V(\bar{r})} \rangle = exp\left\{ \sum_{k=1}^{\infty} \frac{\beta^k}{k!} C_k \right\} \tag{9}$$

In Equation 9, the first two cumulants are given by:

$$C_1 = \langle \Delta V \rangle, \tag{10}$$

$$C_2 = \langle \Delta V^2 \rangle - \langle \Delta V \rangle^2 = \sigma_{\Delta V}^2.$$

Cumulant expansion to the second order provides a more accurate reweighting[7] as the boost potential obtained from PPI-GaMD simulations follows a near-Gaussian distribution[5]. The reweighted free energy $F(A) = -k_B T \ln p(A)$ is calculated as:

$$F(A) = F^*(A) - \sum_{k=1}^{2} \frac{\beta^k}{k!} C_k + F_c \tag{11}$$

where $F^*(A) = -k_B T \ln p^*(A)$ is the modified free energy obtained from PPI-GaMD simulation and F_c is a constant.

S3. Binding free energy calculation using Molecular mechanics-generalized born surface area (MM-GBSA) method

The MM-GBSA method was utilized to calculate the binding free energies of all simulated AF9-peptide complexes. MM-GBSA is an end-point approach that estimates the binding free energy from a single trajectory of the solvated protein–ligand or protein–protein complex. For these calculations, the gmx_MMPBSA software[8] was employed, which integrates AMBER's cpptraj and MMPBSA.py tools. To compute the binding free energy, 100 frames were extracted from the last 50 ns of the trajectory, and the contribution of each residue to the complex was also analyzed over this period. The average values and standard deviations across these frames were

reported. Before performing the calculations with gmx_MMPBSA, the periodic boundary conditions (PBC) were removed from the GROMACS output trajectory. The binding free energy (BE) of a complex can be expressed as:

$$\Delta G_{BE} = G_{\text{AF9-Pep}} - (G_{\text{AF9}} - G_{\text{Pep}}) \quad (12)$$

which can also be given as:

$$\Delta G_{BE} = \Delta E_{\text{MM}} + \Delta G_{\text{Solv}} - T\Delta S \quad (13)$$

The molecular mechanics energy term (ΔE_{MM}) is the combination of bonded (bonds, angles, dihedrals) and non-bonded (van der Waals and electrostatic) energy terms in the gas phase and was computed with the CHARMM36m force field[9].

$$\Delta E_{\text{MM}} = (\Delta E_{\text{Bonds}} + \Delta E_{\text{Angles}} + \Delta E_{\text{Dihedrals}}) + \Delta E_{\text{vdW}} + \Delta E_{\text{Electro}} \quad (14)$$

ΔG_{Solv} comprises of polar and non-polar solvation free energy components.

$$\Delta G_{\text{Solv}} = \Delta G_{\text{Polar}} + \Delta G_{\text{Non-polar}} \quad (15)$$

The polar contribution to the solvation free energy (ΔG_{Polar}) was calculated using the GB/OBC (Generalized Born/Optimized Born Chain) model with igb = 5, whereas the non-polar solvation free energy ($\Delta G_{\text{Non-polar}}$) was determined using the following equation:

$$\Delta G_{\text{SA}} = \gamma \cdot \Delta\text{SASA} + \beta \quad (16)$$

ΔSASA represents the change in solvent-accessible surface area upon complex formation, with empirical constants $\gamma = 0.0072$ kcal·Å^{-2}·mol^{-1} and $\beta = 0$, as defined for the GB models. Entropic contributions were omitted in this study, since the focus is on comparing relative binding free energies. Consequently, the total binding free energy can be represented as the sum of the individual energy components:

$$\Delta G_{BE} = (\Delta E_{\text{Bonds}} + \Delta E_{\text{Angles}} + \Delta E_{\text{Dihedrals}}) + \Delta E_{\text{vdW}} + \Delta E_{\text{Electro}} + \Delta G_{\text{Polar}} + \Delta G_{\text{Non-polar}} \quad (17)$$

To quantify the hydrophobic contributions to the total interaction energy in each complex, the van der Waals energy and nonpolar solvation free energy associated with interfacial hydrophobic residues were summed, as described by the following equations:

$$\Delta E_{Hydrophobic} = \Delta E_{vdW} + \Delta G_{Non-polar} \quad (18)$$

Supplementary Tables

Table S1: Binding free energies and energy components obtained from MM-GBSA analysis of AF9–BCOR, AF9–CBX8, and AF9–DOT1L complexes. The energies are in kcal/mol.

AF9 complexes	EEL	vdW	GB	Surf	Total binding free energy (kcal/mol)
**BCOR**	-256.96	-82.89	268.04	-12.9	-84.72
**CBX8**	-264.33	-87.81	270.53	-12.84	-94.45
**DOT1L**	-452.8	-103.73	459.86	-16.32	-112.99

Table S2: Per-residue decomposition of the total binding free energy and individual energy components from MM-GBSA analysis of AF9-BCOR complex.

Residue	van der Waals	Electrostatic	Polar Solvation	Non-Polar Solv.	TOTAL
	**Avg.**	**Avg.**	**Avg.**	**Avg.**	**Avg.**
**L504**	-1.59	-1.20	1.54	-0.34	-1.59
**L507**	-0.82	-0.89	0.92	-0.02	-0.81
**V508**	-1.12	-0.90	1.15	-0.21	-1.07
**H511**	-2.24	-2.31	2.99	-0.38	-1.94
**L514**	-1.07	-0.01	0.32	-0.13	-0.89
**R518**	-0.19	-7.17	7.46	-0.02	0.08
**R520**	-2.90	-5.41	6.96	-0.57	-1.92
**L523**	-2.02	-0.54	0.99	-0.32	-1.89
**Q524**	-2.25	-2.24	3.11	-0.45	-1.82
**I526**	-0.56	-0.16	0.16	-0.03	-0.59
**V527**	-1.54	0.30	-0.02	-0.27	-1.53
**H535**	-0.08	-0.64	0.85	0.00	0.13
**I538**	-0.52	-0.07	0.25	-0.11	-0.46
**T541**	-1.93	-3.42	3.55	-0.57	-2.37
**T542**	-2.46	-1.38	1.37	-0.33	-2.80
**F543**	-2.68	-3.94	2.68	-0.34	-4.29
**D544**	-0.48	-34.60	32.48	-0.31	-2.91
**F545**	-3.36	-3.80	4.12	-0.35	-3.39
**D546**	-1.08	-32.09	30.09	-0.41	-3.50
**L547**	-2.20	-3.04	2.00	-0.12	-3.36
**C548**	-2.45	-2.21	2.08	-0.36	-2.94
**S549**	-0.39	0.24	0.20	-0.03	0.02
**D551**	-0.02	6.94	-6.78	0.00	0.15

**S1180**	0.00	0.04	-0.02	0.00	0.02
**N1181**	-0.03	-0.02	0.07	-0.01	0.01
**N1193**	-2.54	-7.88	8.45	-0.43	-2.41
**L1194**	-6.26	-0.86	2.27	-0.96	-5.81
**K1195**	-0.72	-116.99	108.86	-0.65	-9.51
**V1196**	-5.68	0.03	0.21	-0.91	-6.35
**C1197**	-3.07	-2.60	3.38	-0.43	-2.71
**I1198**	-5.09	-2.71	2.24	-0.91	-6.48
**E1199**	-2.19	10.00	-7.86	-0.26	-0.32
**L1200**	-3.45	-3.40	3.20	-0.71	-4.36
**T1201**	-1.09	-1.61	2.46	-0.25	-0.49
**G1202**	-0.93	-1.93	2.71	-0.21	-0.36
**L1203**	-3.86	-2.10	2.67	-0.61	-3.90
**P1205**	-0.60	0.70	-0.54	-0.10	-0.54

Table S3: Per-residue decomposition of the total binding free energy and individual energy components from MM-GBSA analysis of AF9-CBX8 complex.

**Residue**	**van der Waals**	**Electrostatic**	**Polar Solvation**	**Non-Polar Solv.**	**TOTAL**
	**Avg.**	**Avg.**	**Avg.**	**Avg.**	**Avg.**
**G329**	-0.07	-0.73	0.91	0.00	0.11
**L333**	-5.86	-5.70	4.51	-1.10	-8.15
**I334**	-3.24	-5.41	4.79	-0.38	-4.24
**A335**	-3.71	-1.53	1.19	-0.64	-4.68
**R336**	-3.20	-63.63	59.10	-0.64	-8.37
**I337**	-5.17	-3.66	2.81	-0.91	-6.93
**P338**	-2.28	-1.65	2.07	-0.16	-2.03
**V339**	-3.03	-4.29	3.98	-0.61	-3.95
**R341**	-4.21	-17.52	20.57	-0.84	-2.00
**I342**	-4.51	-5.68	6.34	-0.89	-4.74
**K501**	-0.16	6.48	-6.03	-0.04	0.25
**L507**	-0.77	0.50	-0.44	-0.03	-0.75
**V508**	-0.76	0.14	0.07	-0.14	-0.69
**H511**	-2.08	-1.60	2.37	-0.38	-1.70
**M515**	-2.61	-2.75	2.46	-0.55	-3.46
**R520**	-3.25	-38.06	39.04	-0.56	-2.83
**L523**	-2.73	-1.17	1.60	-0.44	-2.73
**Q524**	-1.27	0.40	0.53	-0.19	-0.53

**V527**	-0.83	-0.78	0.90	-0.09	-0.79
**I538**	-0.07	-0.78	0.83	0.00	-0.02
**T542**	-3.38	-0.28	0.40	-0.44	-3.71
**F543**	-3.04	-5.61	5.01	-0.50	-4.14
**D544**	-0.99	-32.84	31.28	-0.37	-2.93
**F545**	-2.57	-3.60	3.37	-0.29	-3.09
**D546**	-1.58	-31.66	31.89	-0.42	-1.77
**L547**	-2.07	-2.06	1.15	-0.09	-3.07
**C548**	-2.94	-0.76	0.86	-0.36	-3.19

Table S4: Per-residue decomposition of the total binding free energy and individual energy components from MM-GBSA analysis of AF9-DOT1L complex.

**Residue**	**van der Waals**	**Electrostatic**	**Polar Solvation**	**Non-Polar Solv.**	**TOTAL**
	**Avg.**	**Avg.**	**Avg.**	**Avg.**	**Avg.**
**L504**	-0.88	-0.25	0.49	-0.25	-0.90
**L507**	-0.79	0.02	0.10	-0.03	-0.70
**V508**	-0.80	-0.18	0.39	-0.13	-0.73
**H511**	-2.41	-1.83	2.41	-0.39	-2.23
**L514**	-1.33	0.47	-0.04	-0.17	-1.08
**M515**	-2.33	-0.41	0.50	-0.38	-2.63
**R518**	-0.47	19.02	-18.44	-0.07	0.04
**R520**	-2.69	16.81	-15.18	-0.61	-1.67
**L523**	-2.44	-1.26	1.75	-0.39	-2.33
**Q524**	-2.24	-3.80	3.90	-0.45	-2.59
**V527**	-2.26	-1.43	1.79	-0.37	-2.26
**N528**	-1.60	-1.36	2.65	-0.21	-0.52
**E531**	-1.75	-80.10	83.03	-0.78	0.40
**I538**	-1.57	0.28	-0.03	-0.35	-1.67
**T541**	-1.27	-1.87	2.30	-0.25	-1.09
**T542**	-2.64	-1.27	0.88	-0.32	-3.34
**F543**	-3.21	-4.55	3.91	-0.41	-4.26
**D544**	-0.72	-38.86	38.97	-0.26	-0.87
**F545**	-2.62	-2.49	2.95	-0.32	-2.48
**D546**	-1.54	-55.29	55.90	-0.41	-1.35
**L547**	-2.35	-1.57	0.81	-0.11	-3.22
**C548**	-2.64	1.90	-1.64	-0.36	-2.74

**N877**	-1.28	1.53	0.02	-0.26	0.00
**K878**	-1.65	-55.29	53.54	-0.25	-3.65
**L879**	-5.55	-5.63	4.15	-1.04	-8.08
**P880**	-2.51	-5.61	5.46	-0.31	-2.98
**V881**	-5.99	-0.99	0.92	-0.97	-7.02
**S882**	-2.10	-13.05	10.12	-0.57	-5.60
**I883**	-4.92	-3.42	2.38	-0.92	-6.87
**P884**	-2.92	-0.18	1.57	-0.44	-1.97
**L885**	-3.73	-2.56	2.65	-0.58	-4.21
**S887**	-1.10	0.33	0.86	-0.19	-0.10
**V888**	-3.30	-2.58	2.43	-0.49	-3.94
**V889**	-1.08	-1.68	1.35	-0.23	-1.64
**L890**	-2.42	1.40	-1.12	-0.38	-2.52
**P891**	-4.46	-6.42	6.30	-0.84	-5.41

Table S5: AF9-BCOR interactions observed from MOE.

S.No.	**Interactions**	**Energy (kcal/mol)**
1	His511-Lys1195	-1.4
2	Thr541-Leu1200	-3.9
3	Thr542-Ile1198	-0.5
4	Phe543-Ile1198	-3.8
5	Asp544-Val1196	-0.5
6	**Phe545-Val1196**	**-5**
7	Leu547-Leu1194	-2.6

Table S6: AF9-CBX8 interactions observed from MOE.

**S.No.**	**Interactions**	**Energy (kcal/mol)**
**1**	**Phe543-Ile337**	**-5.3**
**2**	Phe545-Ala335	-4.2
**3**	Leu547-Leu333	-4.4

Table S7: AF9-DOT1L interactions observed from MOE.

S.No.	Interactions	Energy (kcal/mol)
**1**	His511-Pro880	-0.90
**2**	Gln524-Val889	-0.60
**3**	**Phe543-Ile883**	**-2.5**
**4**	Asp544-Val881	-0.50
**5**	Phe545-Val881	-2.3

Table S8: Summary of the PPI-GaMD simulations performed on the AF9 in the presence of BCOR/CBX8/DOT1L.

System Name	System Size	ID	Simulation Length	Boost Potential (kcal/mol)
**AF9-BCOR**	61881	Simulation 1	500 ns	9.89 ± 5.34
		Simulation 2	500 ns	9.00 ± 4.59
		Simulation 3	500 ns	9.70 ± 4.97
		Simulation 4	500 ns	9.47 ± 4.73
		Simulation 5	500 ns	10.38 ± 5.04
**AF9-CBX8**	57502	Simulation 1	500 ns	8.36 ± 3.41
		Simulation 2	500 ns	9.29 ± 3.44
		Simulation 3	500 ns	9.26 ± 3.41
		Simulation 4	500 ns	9.72 ± 3.81
		Simulation 5	500 ns	8.95 ± 3.23
**AF9-DOT1L**	53865	Simulation 1	500 ns	10.03 ± 3.21
		Simulation 2	500 ns	9.42 ± 3.08
		Simulation 3	500 ns	9.97 ± 3.2
		Simulation 4	500 ns	9.04 ± 3.26
		Simulation 5	500 ns	9.55 ± 3.49

Table S9: DPeak clustering analysis of concatenated GaMD trajectory of AF9-BCOR complex.

#Cluster	Frames	Frac	AvgDist	Stdev	Centroid	AvgCDist
**0**	49800	0.02	34.852	12.28	785481	25.249
**1**	22726	0.009	31.415	11.163	774441	29.439
**2**	16529	0.007	20.891	14.237	2030141	20.541
**3**	6419	0.003	24.281	7.591	952601	33.828
**4**	5139	0.002	33.578	13.457	1970821	34.744
**5**	4756	0.002	26.495	9.204	1409841	33.869
**6**	4668	0.002	22.701	7.169	929781	29.135
**7**	3201	0.001	21.414	6.226	1863681	34.079
**8**	2354	0.001	2.956	0.781	1002864	22.451
**9**	1827	0.001	14.17	8.098	538021	21.027
**10**	1666	0.001	17.952	5.679	1416881	30.917
**11**	1323	0.001	13.204	11.207	2110581	22.769
**12**	1203	0	19.294	10.893	86861	24.621
**13**	1091	0	0	0	81179	23.041
**14**	842	0	2.58	0.86	2093697	21.441
**15**	772	0	7.513	4.47	1048141	21.023
**16**	746	0	19.782	7.24	1716181	31.016
**17**	652	0	16.636	4.666	1119301	34.076
**18**	557	0	19.163	5.856	1868581	39.993
**19**	545	0	4.182	1.297	2095505	20.716
**20**	530	0	13.68	4.416	1746501	25.339
**21**	495	0	5.142	2.158	72297	21.493
**22**	378	0	3.969	1.231	2097173	21.413
**23**	326	0	6.908	5.209	79803	22.813
**24**	307	0	4.681	1.386	2124616	21.545
**25**	293	0	14.854	4.111	1708261	35.932
**26**	279	0	3.19	1.012	1057420	23.35
**27**	272	0	14.66	4.751	1329621	37.099
**28**	165	0	4.455	1.587	2092687	21.518
**29**	137	0	4.384	1.459	1053658	22.263
**30**	57	0	13.906	5.193	838841	38.522
**31**	57	0	11.188	3.535	2474381	33.077
**32**	14	0	7.237	2.529	1329081	35.472

Table S10: DPeak Clustering analysis of concatenated GaMD trajectory of AF9-CBX8 complex.

#Cluster	Frames	Frac	AvgDist	Stdev	Centroid	AvgCDist
**0**	16989	0.007	8.561	8.728	1506861	13.802
**1**	11880	0.005	3.755	1.32	49805	15.008
**2**	11095	0.004	4.224	1.151	534357	14.719
**3**	9139	0.004	3.638	1.145	2068253	14.245
**4**	9040	0.004	4.874	2.283	2209334	14.173
**5**	8829	0.004	4.879	1.781	2193650	14.174
**6**	6579	0.003	4.102	1.271	1182641	14.522
**7**	6198	0.002	2.921	1.19	1258434	14.359
**8**	5958	0.002	22.632	10.319	1425501	20.68
**9**	5258	0.002	3.845	1.383	2233485	14.597
**10**	5136	0.002	2.419	0.651	41956	14.664
**11**	5124	0.002	24.526	9.084	395301	24.204
**12**	4956	0.002	4.378	1.866	1276862	15.15
**13**	4954	0.002	20.089	8.485	240021	22.394
**14**	4761	0.002	8.489	4.432	110941	15.323
**15**	4614	0.002	3.195	0.864	1211167	14.07
**16**	4273	0.002	4.634	1.504	1069768	14.75
**17**	4216	0.002	8.297	6.722	2100351	14.676
**18**	4201	0.002	4.772	1.519	649838	14.701
**19**	3585	0.001	4.294	1.649	1083164	14.149
**20**	3441	0.001	3.124	0.943	1238998	14.108
**21**	3427	0.001	4.186	1.361	1041159	14.17
**22**	3403	0.001	22.501	8.589	2458881	23.733
**23**	3403	0.001	2.344	0.746	2258212	14.733
**24**	3327	0.001	2.871	0.794	1267816	14.31
**25**	3103	0.001	20.6	12.238	1715161	17.575
**26**	3046	0.001	24.152	7.911	245001	26.839
**27**	2935	0.001	3.75	1.787	2057200	14.254
**28**	2786	0.001	6.113	1.879	2318147	14.024
**29**	2573	0.001	4.106	1.167	754634	15.959
**30**	2388	0.001	24.223	8.669	871321	25.423
**31**	2372	0.001	15.9	8.165	1766701	20.028
**32**	2310	0.001	2.782	0.845	2222288	14.44
**33**	2307	0.001	2.512	0.642	1395005	20.065
**34**	2244	0.001	18.57	9.139	1952681	19.825
**35**	2230	0.001	5.138	1.43	668971	15.15

**36**	2182	0.001	6.833	2.231	1364881	19.003
**37**	2107	0.001	6.538	2.23	1663999	14.723
**38**	1981	0.001	20.22	10.082	454301	21.566
**39**	1947	0.001	23.327	7.38	1913941	33.49
**40**	1844	0.001	5.174	2.626	546537	13.777
**41**	1659	0.001	12.889	5.628	1800781	23.187
**42**	1635	0.001	3.649	1.031	673088	15.68
**43**	1580	0.001	5.22	2.151	795391	16.021
**44**	1570	0.001	6.969	2.507	1702278	15.66
**45**	1517	0.001	5.367	1.661	2137877	14.706
**46**	1391	0.001	3.704	1.602	1245813	14.741
**47**	1352	0.001	18.498	6.461	899861	26.634
**48**	1223	0	3.949	1.19	693597	14.787
**49**	1156	0	19.061	8.67	381061	23.11
**50**	1154	0	8.594	4.393	1314061	16.487
**51**	1102	0	4.791	1.413	828795	15.037
**52**	1091	0	4.186	1.805	2450303	16.486
**53**	1088	0	5.759	2.285	1643291	14.426
**54**	927	0	3.85	1.333	2120651	16.101
**55**	909	0	10.943	6.414	1673421	17.143
**56**	893	0	14.657	9.345	858241	18.24
**57**	885	0	21.386	9.341	1492921	22.41
**58**	855	0	7.139	2.549	2399141	15.58
**59**	830	0	5.356	1.818	1384825	18.971
**60**	826	0	6.233	1.821	2124361	14.922
**61**	816	0	4.278	1.211	1387234	19.083
**62**	798	0	11.747	6.517	497781	19.019
**63**	731	0	7.204	2.679	1792081	19.046
**64**	696	0	6.936	3.083	1375288	17.844
**65**	596	0	16.541	5.593	397361	26.848
**66**	541	0	10.725	7.017	1823881	22.833
**67**	533	0	6.22	2.131	2355796	15.45
**68**	388	0	3.228	0.979	226162	18.64
**69**	370	0	3.925	1.383	1118125	17.485
**70**	352	0	2.892	0.931	1117689	18.308
**71**	278	0	14.08	6.961	379881	24.033
**72**	255	0	9.968	4.251	461521	21.737
**73**	231	0	5.022	1.674	1739781	17.865
**74**	215	0	4.959	2.719	2452824	15.511

**75**	163	0	9.194	4.573	495161	18.689
**76**	47	0	10.398	4.63	271521	45.177
**77**	38	0	4.268	0	288607	46.378
**78**	25	0	12.121	4.775	287741	42.841
**79**	13	0	9.169	4.362	288561	44.948
**80**	13	0	6.326	2.724	397801	24.234

Table S11: DPeak clustering analysis of concatenated trajectory of AF9-DOT1L complex.

#Cluster	Frames	Frac	AvgDist	Stdev	Centroid	AvgCDist
**0**	34050	0.014	5.332	3.293	379462	16.587
**1**	10598	0.004	5.549	1.968	939511	15.865
**2**	7496	0.003	3.596	1.32	2118271	15.83
**3**	6842	0.003	5.725	2.14	787106	16.038
**4**	6686	0.003	8.346	4.063	1199761	16.15
**5**	6160	0.002	3.288	1.351	257247	15.597
**6**	5856	0.002	6.893	3.559	2125278	15.728
**7**	4660	0.002	7.009	2.428	1323861	16.909
**8**	4524	0.002	7.061	2.226	1129541	16.247
**9**	4401	0.002	6.759	2.098	1003329	15.907
**10**	4006	0.002	26.59	10.864	2441421	25.897
**11**	4002	0.002	2.553	0.828	870006	16.049
**12**	3779	0.002	5.139	1.676	1651204	15.745
**13**	3748	0.001	25.884	10.512	2406721	24.587
**14**	3613	0.001	4.565	2.008	288748	15.815
**15**	3487	0.001	4.797	1.984	971982	15.902
**16**	3476	0.001	7.449	2.259	1493241	16.245
**17**	3217	0.001	5.709	1.68	1621396	17.508
**18**	3190	0.001	8.235	3.027	1487881	15.306
**19**	3151	0.001	4.281	2.049	860779	15.481
**20**	3074	0.001	7.993	7.425	886836	16.511
**21**	2888	0.001	3.888	1.343	2074475	15.884
**22**	2794	0.001	3.49	1.405	196659	15.43
**23**	2593	0.001	4.849	1.746	568196	16.678
**24**	2377	0.001	5.87	2.142	530664	15.661
**25**	2296	0.001	7.169	2.429	1357229	17.071
**26**	2131	0.001	3.866	1.539	584550	15.95
**27**	2126	0.001	8.385	3.935	16161	15.397
**28**	2079	0.001	6.142	2.071	876836	15.644

**29**	1963	0.001	4.789	1.498	1529376	17.051
**30**	1952	0.001	3.893	1.253	1630665	16.69
**31**	1906	0.001	3.524	1.406	1695498	15.762
**32**	1900	0.001	4.921	2.257	48947	17.534
**33**	1884	0.001	20.406	10.489	1721561	20.543
**34**	1854	0.001	4.462	1.417	210068	15.258
**35**	1776	0.001	6.172	1.783	1670901	15.393
**36**	1705	0.001	7.569	2.473	121641	15.991
**37**	1605	0.001	4.954	1.509	987529	15.191
**38**	1555	0.001	10.553	3.432	1760601	19.409
**39**	1456	0.001	12.74	10.747	725361	16.244
**40**	1419	0.001	5.531	1.731	1178774	15.276
**41**	1263	0.001	6.948	2.293	243381	15.906
**42**	1227	0	13.873	9.819	1419221	19.261
**43**	1190	0	16.835	9.828	2472381	18.393
**44**	1136	0	4.163	1.478	224553	15.35
**45**	1119	0	15.951	9.662	2266301	18.884
**46**	1078	0	4.74	1.75	191293	15.25
**47**	1068	0	6.3	1.827	252401	16.535
**48**	1067	0	5.589	3.34	1557388	16.816
**49**	983	0	3.302	1.176	1613406	17.253
**50**	974	0	6.474	2.366	1804110	20.181
**51**	962	0	8.132	3.089	2228681	17.548
**52**	957	0	7.514	2.557	2220821	17.883
**53**	922	0	4.255	1.38	1341926	16.277
**54**	903	0	19.442	9.873	2379821	22.532
**55**	902	0	1.455	0.363	1508932	15.655
**56**	867	0	27.859	11.484	1909041	29.532
**57**	857	0	5.511	2.304	73984	17.281
**58**	766	0	16.991	7.245	1852741	20.373
**59**	764	0	18.582	6.826	2421461	30.906
**60**	651	0	14.477	6.472	2429921	22.182
**61**	642	0	14.44	7.169	2452761	18.248
**62**	608	0	23.433	9.275	1911401	34.529
**63**	581	0	18.012	7.661	1903241	23.731
**64**	569	0	14.708	5.355	2408301	24.079
**65**	565	0	20.049	7.367	2435821	32.882
**66**	518	0	4.362	1.212	1461146	16.983
**67**	495	0	9.961	6.139	2258281	18.581

**68**	467	0	5.916	1.963	1377610	16.599
**69**	447	0	15.062	9.694	2372321	26.739
**70**	442	0	5.753	2.145	1086826	17.346
**71**	418	0	7.759	2.397	1789241	20.113
**72**	418	0	5.398	1.737	57792	18.757
**73**	399	0	17.633	6.219	1982301	36.693
**74**	362	0	8.084	2.802	1797601	21.145
**75**	320	0	9.097	3.502	1750041	20.152
**76**	305	0	2.456	0.812	1752985	20.787
**77**	233	0	16.393	5.798	2320081	38.954
**78**	219	0	19.388	10.369	1873601	29.299
**79**	193	0	5.34	1.673	2180864	17.948
**80**	155	0	13.221	5.007	1895481	30.572
**81**	121	0	14.946	6.075	1946601	45.011
**82**	33	0	3.975	1.268	2435903	31.632
**83**	20	0	8.667	3.667	1994461	34.916
**84**	17	0	12.319	5.705	1918781	39.064
**85**	16	0	12.233	5.019	2302341	38.782
**86**	13	0	8.715	5.084	1914041	36.391
**87**	6	0	11.91	5.072	1926241	39.826

Table S12: Block-averaged COM distances for AF9–DOT1L PPI-GaMD replicas.

**Replica**	**Block 1 (Å)**	**Block 2 (Å)**	**Block 3 (Å)**	**Block 4 (Å)**
R1	14.61 ± 2.14	14.19 ± 1.50	12.57 ± 1.34	11.66 ± 0.70
R2	14.92 ± 2.34	14.36 ± 2.51	14.00 ± 2.62	16.17 ± 1.26
R3	12.22 ± 2.50	14.33 ± 2.63	15.57 ± 2.32	16.08 ± 2.38
R4	11.52 ± 1.58	15.01 ± 3.79	22.62 ± 9.69	40.82 ± 9.77
R5	13.80 ± 2.19	16.74 ± 2.21	36.10 ± 13.93	22.90 ± 5.16

Table S13: Classification of the clusters obtained from the DPeak clustering into bound, intermediate and unbound states for AF9-peptide complexes.

**S.No.**	**Complex**	**Clusters in Bound state**	**Clusters in Intermediate state**	**Clusters in Unbound state**
**1**	AF9-BCOR	6	9	18
**2**	AF9-CBX8	15	47	19
**3**	AF9-DOT1L	5	61	24

Supplementary Figures

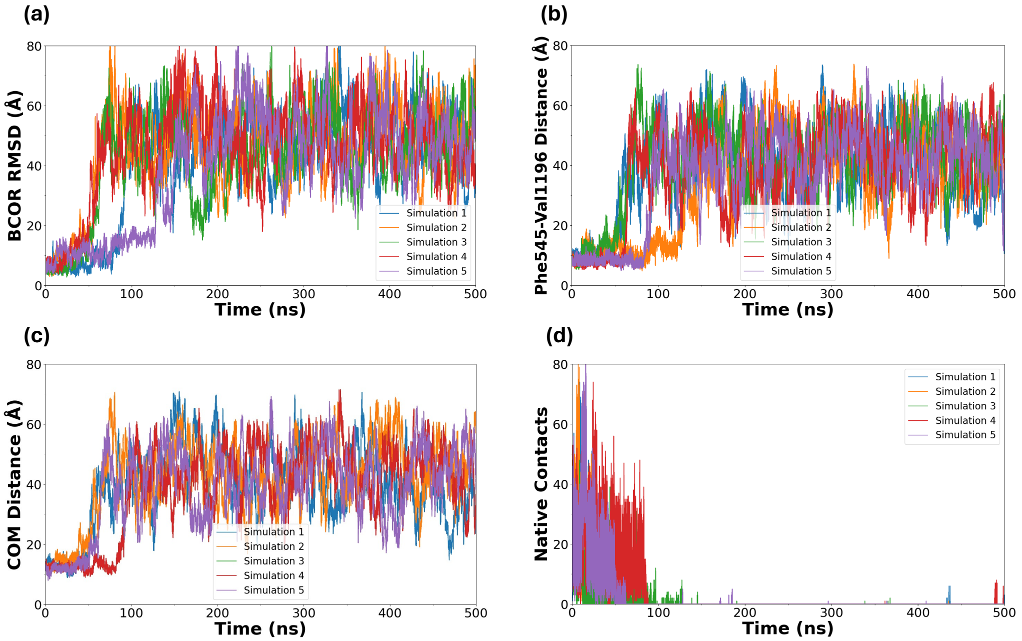

Figure S1: (a) RMSD, (b) F545-V1196 distance, (c) COM distance, and (d) Native contacts between AF9 and BCOR plots of individual simulations for BCOR dissociation from AF9.

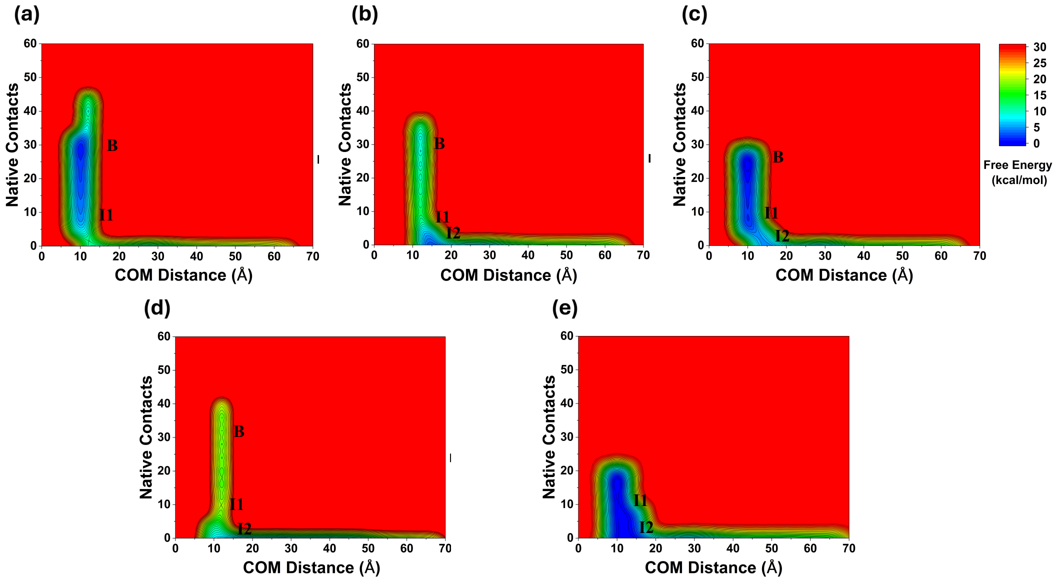

Figure S2: Two-dimensional free energy landscapes obtained from individual PPI-GaMD simulations of the AF9–BCOR complex: (a) simulation 1, (b) simulation 2, (c) simulation 3, (d) simulation 4, and (e) simulation 5, constructed by selecting COM distance and Native contacts as collective variables. The bound and intermediate states are indicated by B, I1, and I2, respectively.

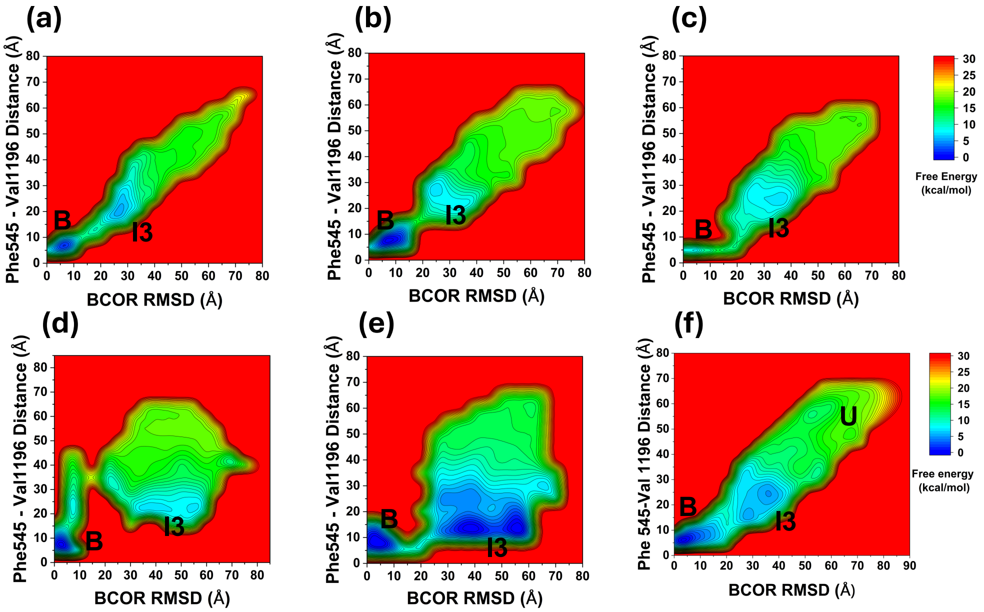

Figure S3: Two-dimensional free energy landscapes obtained from individual PPI-GaMD simulations of the AF9–BCOR complex: (a) simulation 1, (b) simulation 2, (c) simulation 3, (d) simulation 4, and (e) simulation 5, and (f) combined FEL from 5 individual simulations, constructed by selecting BCOR RMSD and F545-V1196 distance as collective variables. The bound, intermediate and unbound states are indicated by B, I3, and U, respectively.

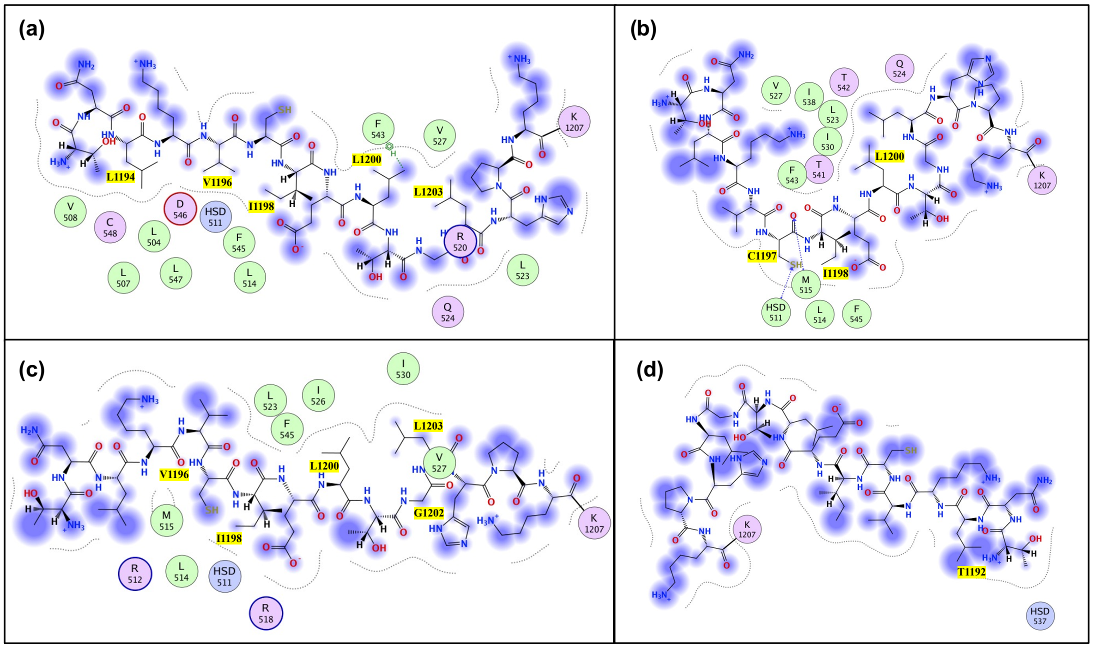

Figure S4: 2D interaction plots of (a) bound, intermediate states (b) I1, (c) I2, and (d) I3, identified from the 2D free energy landscape of AF9-BCOR complex. The residues of BCOR showing hydrophobic interactions with AF9 residues are highlighted in yellow.

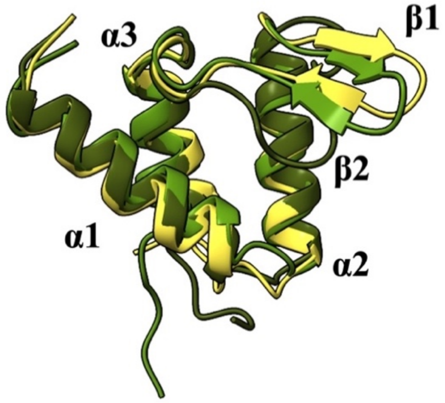

Figure S5: Comparison of AF9 structures in bound, intermediate, and unbound states of the AF9–BCOR complex. AF9 conformations are shown in yellow (bound), light green (intermediate), and dark green (unbound). Secondary structure elements are indicated by Greek letters.

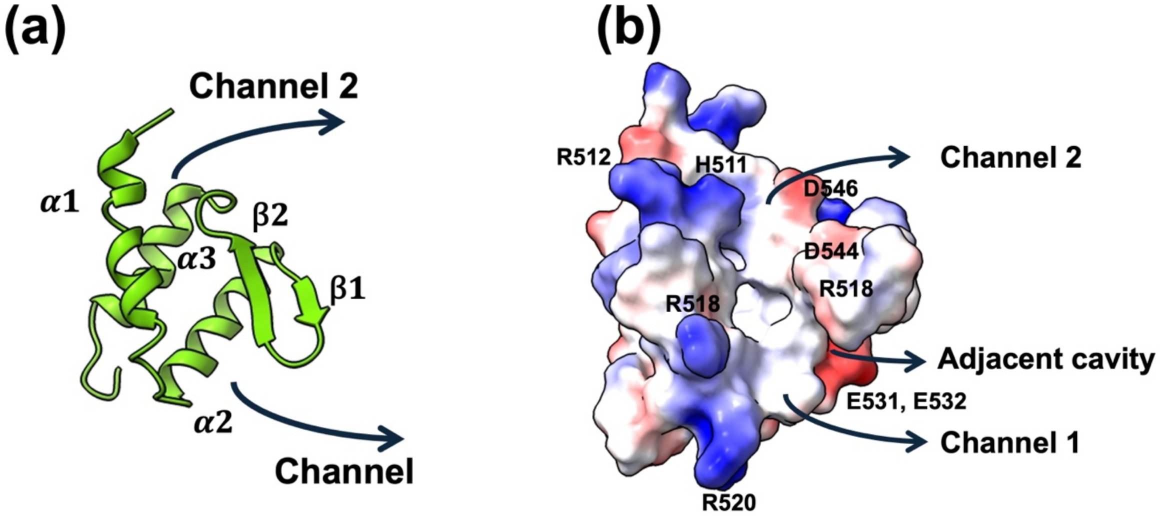

Figure S6: Structural representation of AF9 highlighting two probable peptide dissociation pathways (Channel 1 and Channel 2). (a) Cartoon representation of AF9 secondary structure. (b) Electrostatic surface view of the dissociation sites. Peptide release via Channel 1 can occur either directly or through an adjacent cavity-assisted route.

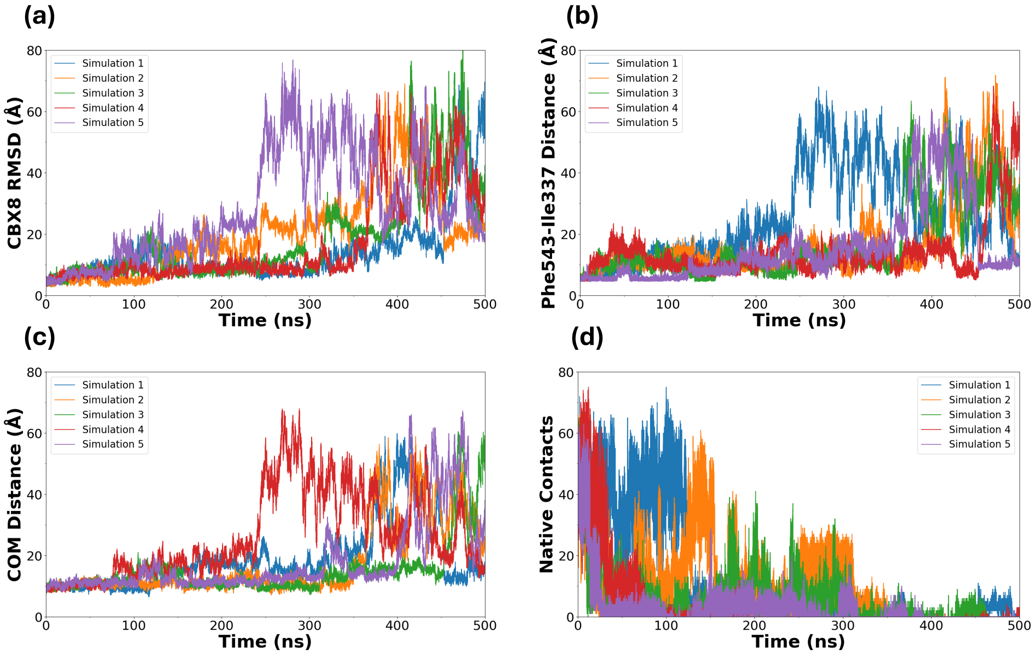

Figure S7: (a) RMSD, (b) F543-I337 distance, (c) COM distance, and (d) Native contacts between AF9 and CBX8 plots of individual simulations for CBX8 dissociation from AF9.

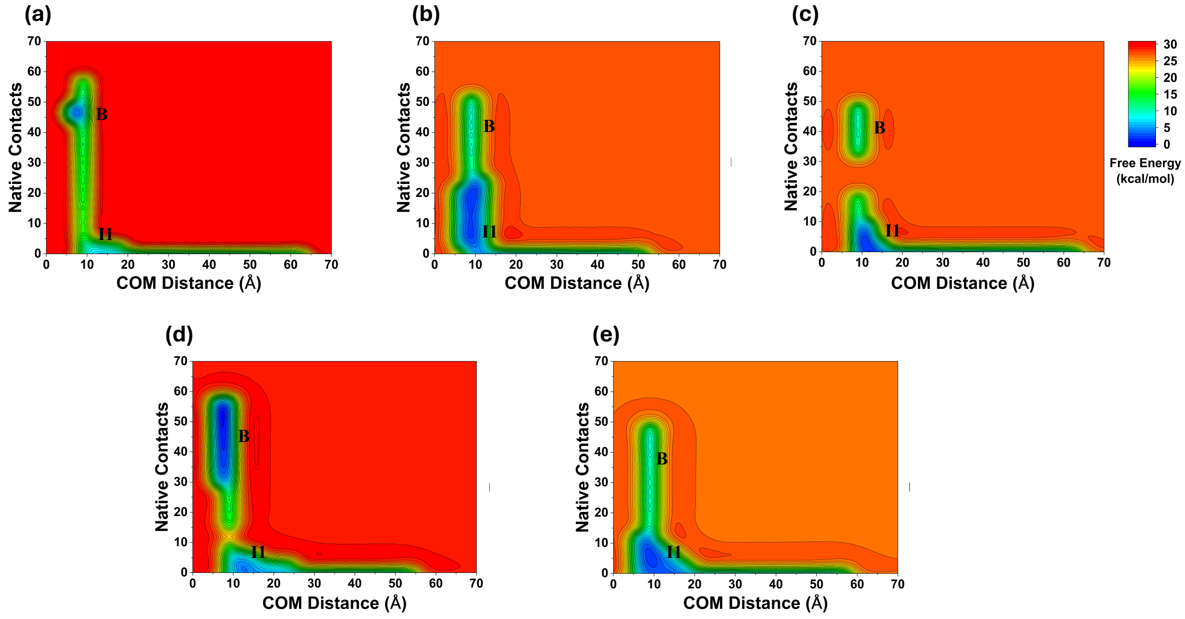

Figure S8: Two-dimensional free energy landscapes obtained from individual PPI-GaMD simulations of the AF9–CBX8 complex: (a) simulation 1, (b) simulation 2, (c) simulation 3, (d) simulation 4, and (e) simulation 5, constructed by selecting COM distance and Native contacts as collective variables. The bound and intermediate states are indicated by B, and I1, respectively.

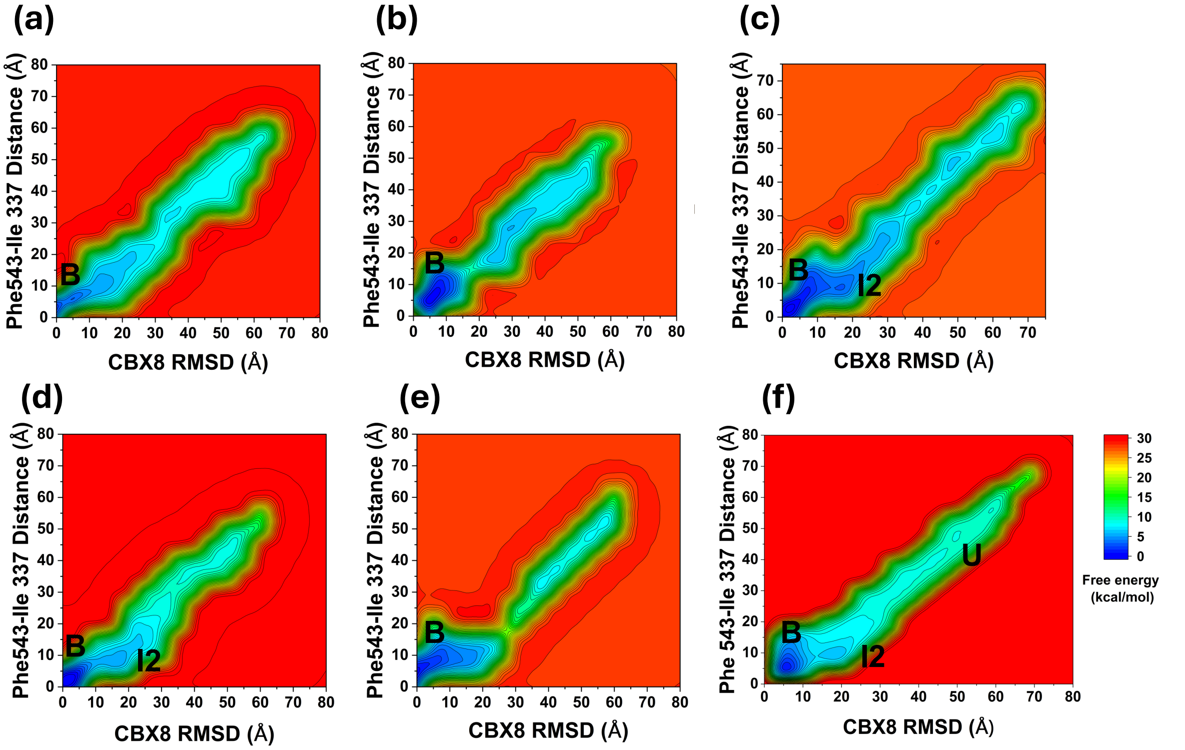

Figure S9: Two-dimensional free energy landscapes obtained from individual PPI-GaMD simulations of the AF9–CBX8 complex: (a) simulation 1, (b) simulation 2, (c) simulation 3, (d) simulation 4, and (e) simulation 5, and (f) combined FEL from 5 individual simulations, constructed by selecting CBX8 RMSD and F543-I337 distance as collective variables. The bound, intermediate and unbound states are indicated by B, I2, and U, respectively.

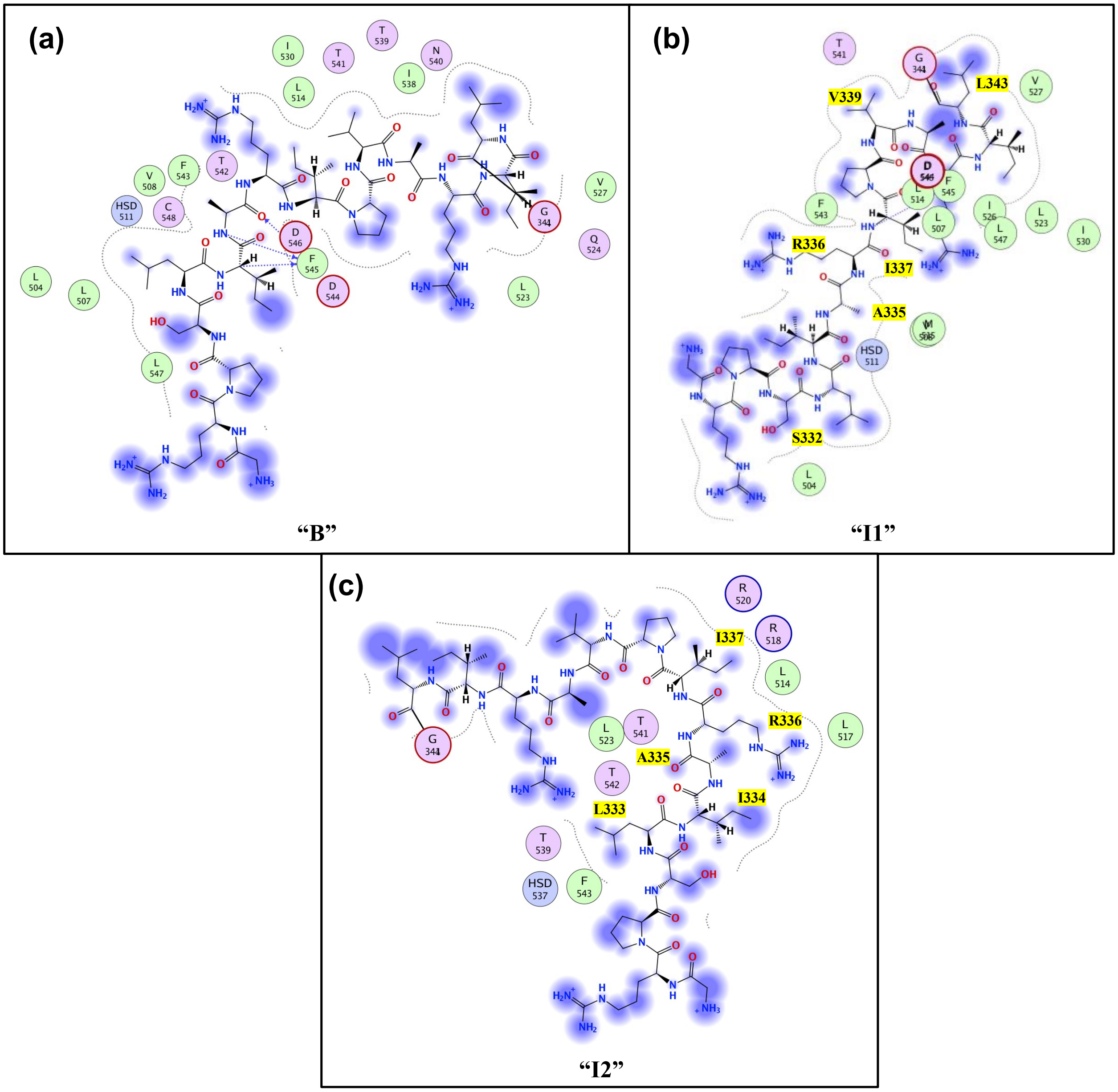

Figure S10: 2D interaction plots of (a) bound and intermediate state (b) I1 and (c) I2 identified from the 2D free energy landscape of AF9-CBX8 complex.

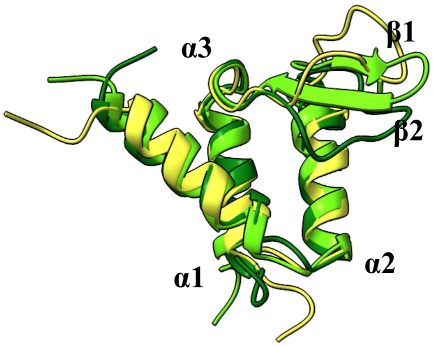

Figure S11: Comparison of AF9 structure in bound, intermediate and unbound forms in AF9-CBX8 complex. AF9 in bound, intermediate, and unbound conformations are shown in yellow, light green, and dark green, respectively.

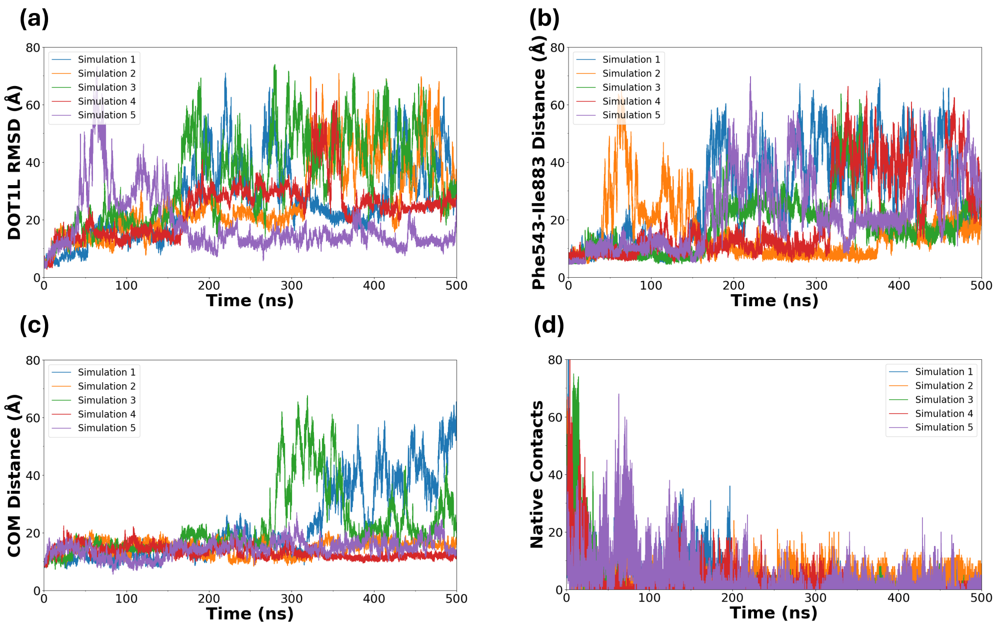

Figure S12: (a) RMSD, (b) F543-I883 distance, (c) COM distance, and (d) Native contacts between AF9 and DOT1L plots of individual simulations for DOT1L dissociation from AF9.

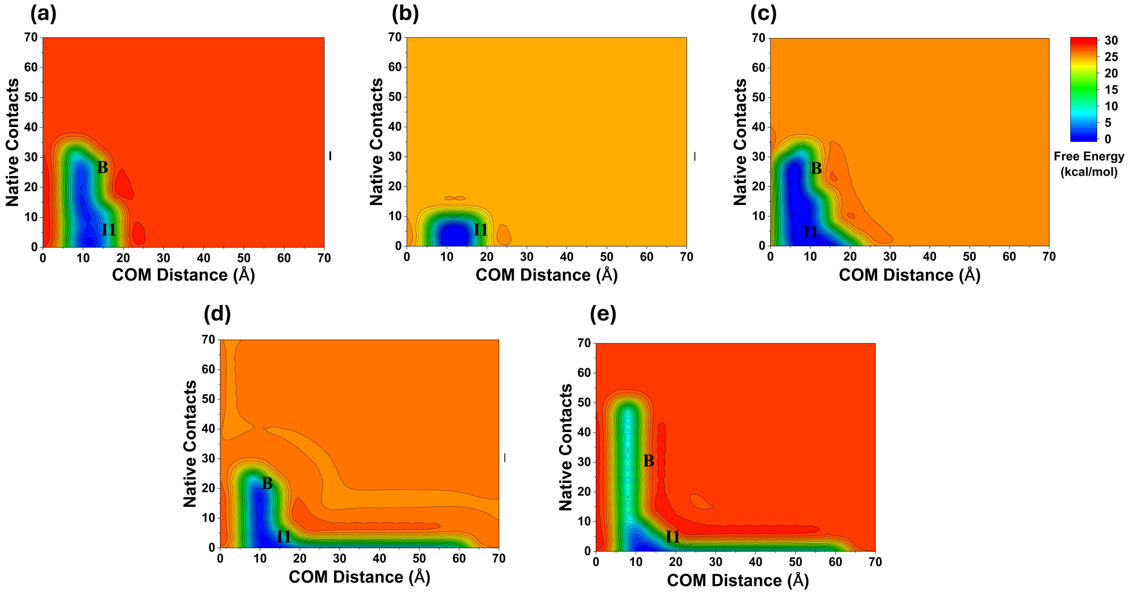

Figure S13: Two-dimensional free energy landscapes obtained from individual PPI-GaMD simulations of the AF9–DOT1L complex: (a) simulation 1, (b) simulation 2, (c) simulation 3, (d) simulation 4, and (e) simulation 5, constructed by selecting COM distance and Native contacts as collective variables. The bound and intermediate states are indicated by B, and I1, respectively.

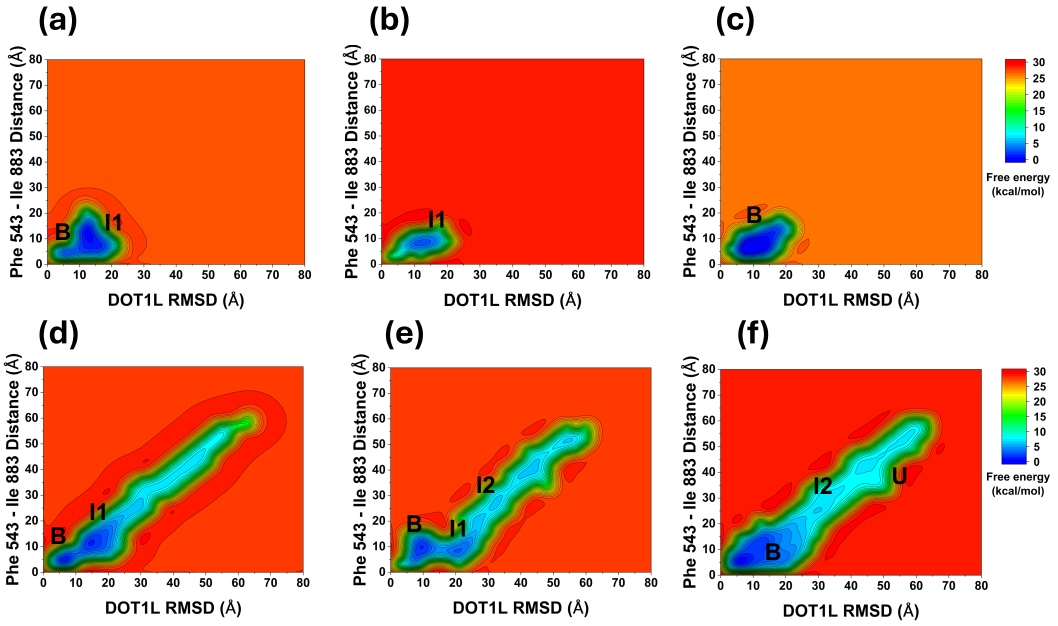

Figure S14: Two-dimensional free energy landscapes obtained from individual PPI-GaMD simulations of the AF9–DOT1L complex: (a) simulation 1, (b) simulation 2, (c) simulation 3, (d) simulation 4, and (e) simulation 5, and (f) combined FEL from 5 individual simulations,

constructed by selecting DOT1L RMSD and F543-I883 distance as collective variables. The bound, intermediate and unbound states are indicated by B, I1, I2, and U, respectively.

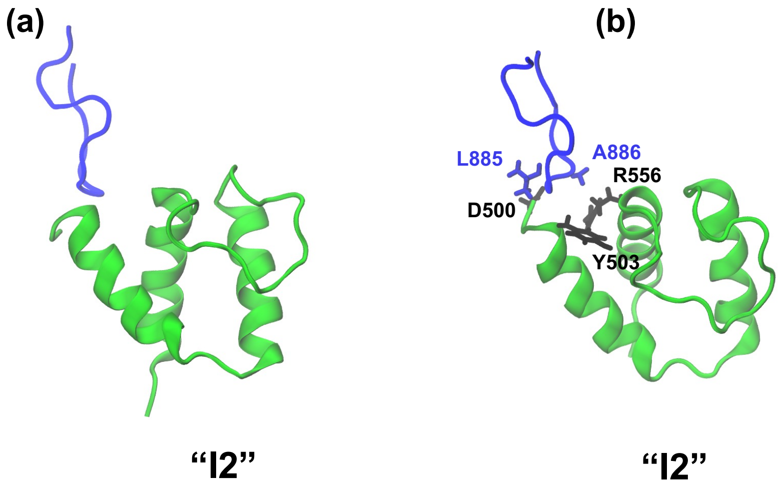

Figure S15: Intermediate I2 structure (cluster 82) and its molecular interactions in AF9-DOT1L complex observed during the dissociation of DOT1L.

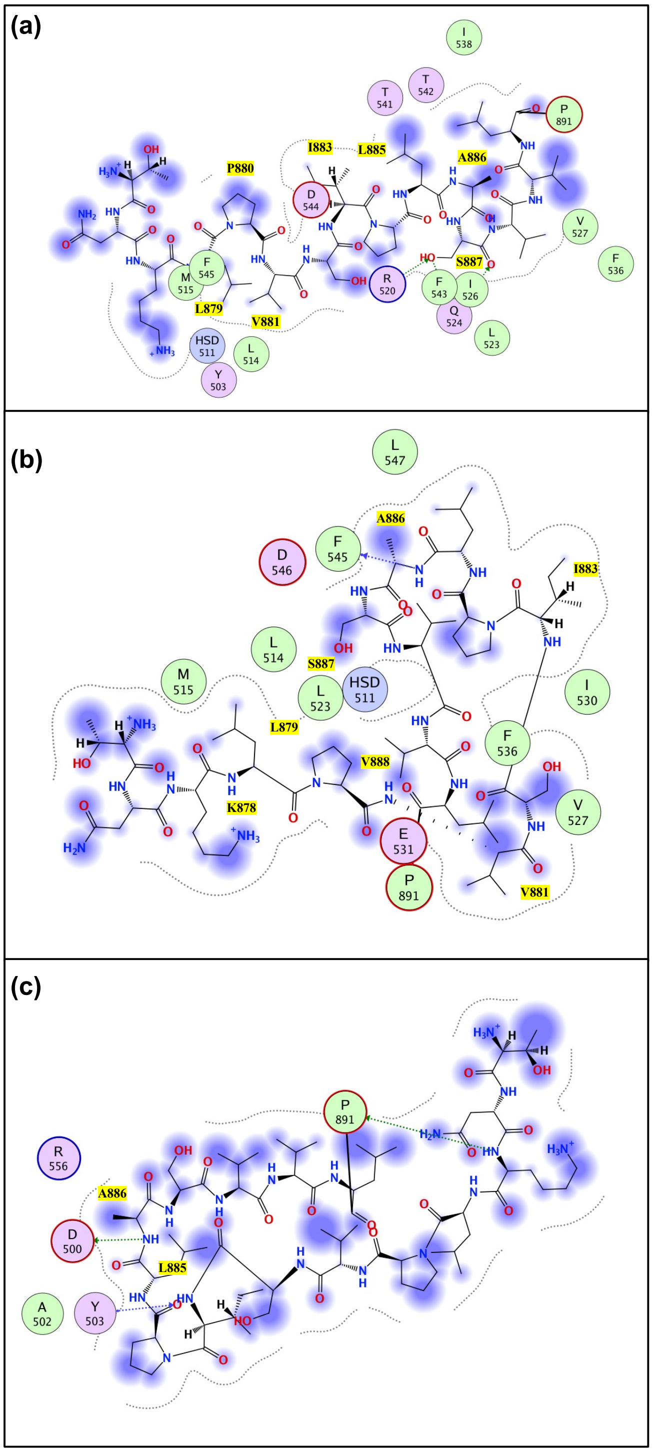

Figure S16: 2D interaction plots of (a) bound, intermediate states (b) I1, and (c) I2, identified from the 2D free energy landscape of AF9-DOT1L complex.

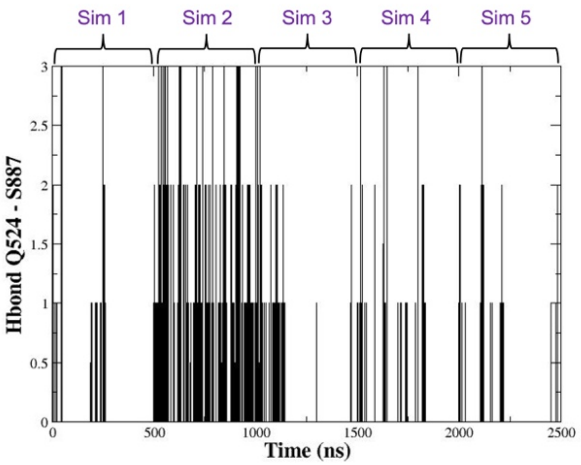

Figure S17: Hbond formation with time between Q524-S887 in GaMD concatenated trajectory of AF9-DOT1L complex.

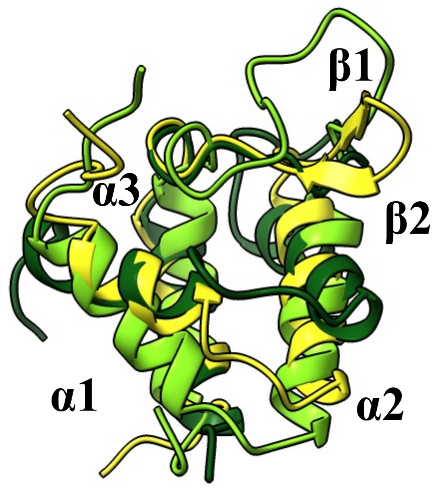

Figure S18: Comparison of AF9 structure in bound, intermediate and most populated forms in AF9-DOT1L complex. AF9 in bound, intermediate 1, and intermediate 2 are shown in yellow, dark green, and light green, respectively.

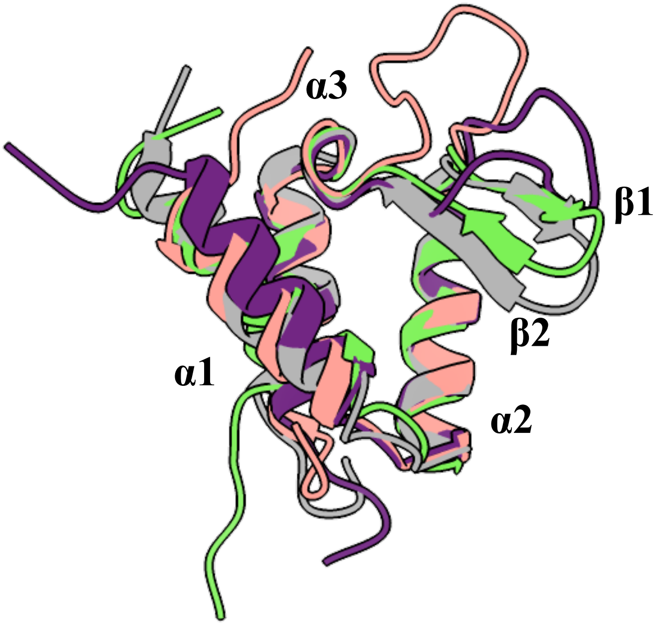

Figure S19: Alignment of intermediate AF9 structures in the BCOR (green), CBX8 (purple), and DOT1L (orange) complexes with the NMR structure (PDB: 6B7G in grey).

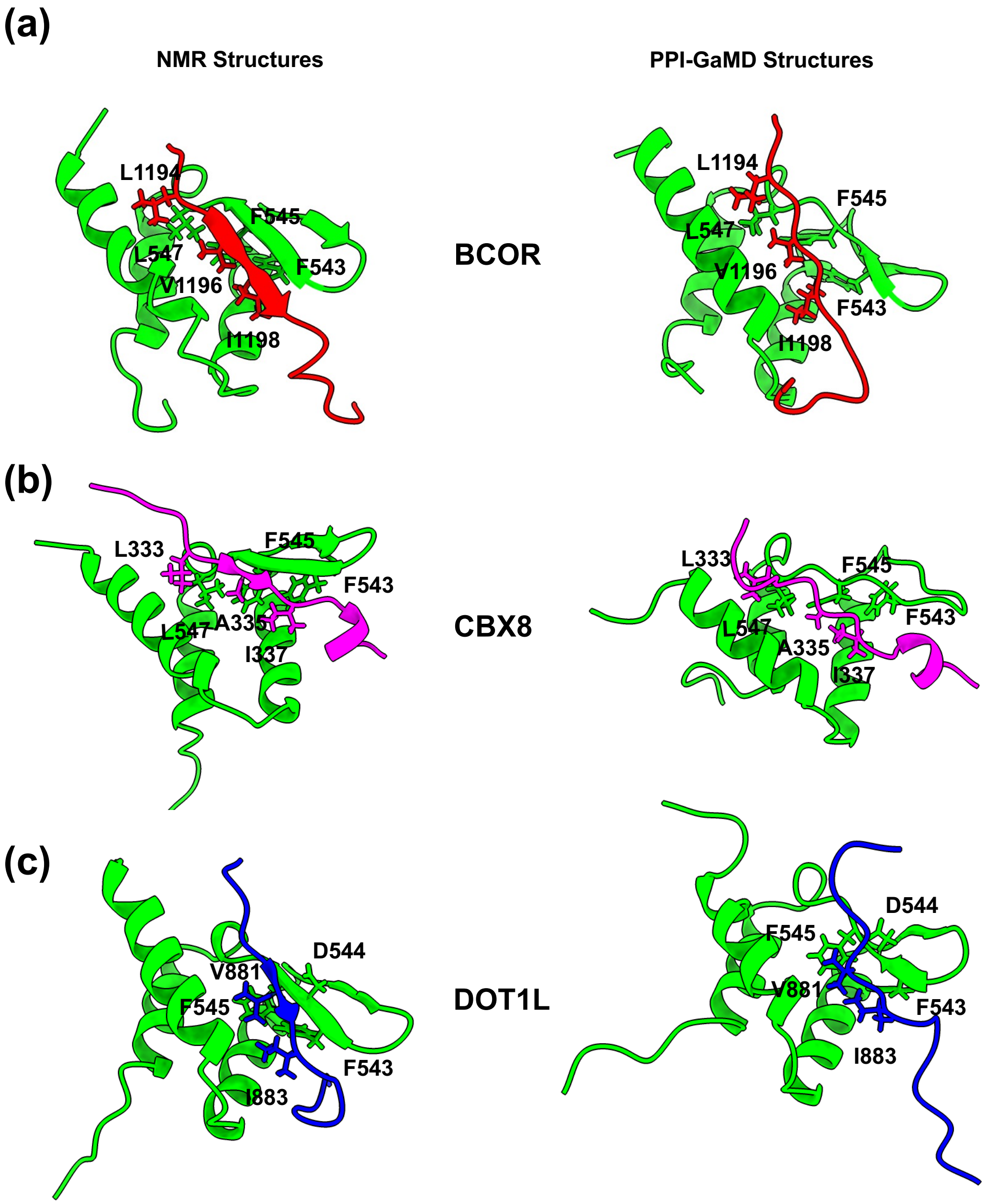

Figure S20: Comparison of key interfacial interactions observed in experimentally determined (a) AF9-BCOR, (b) AF9-CBX8, and (d) AF9-DOT1L complexes and in the bound-state ensemble sampled by PPI-GaMD simulations.

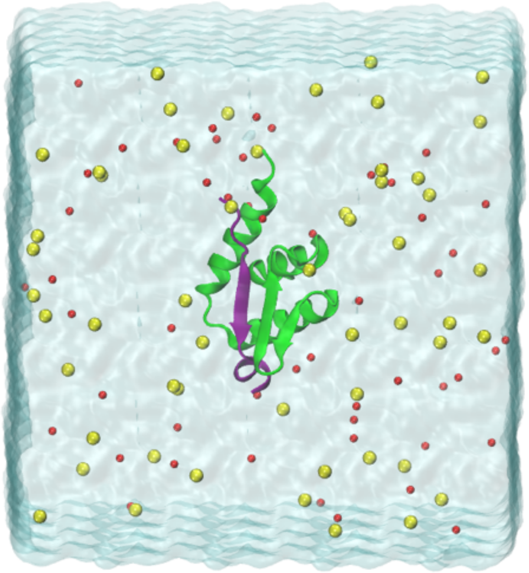

Figure S21: Visualization of prepared water box containing AF9-CBX8 complex, TIP3P water molecules and 0.15 M sodium chloride. 4

REFERENCES-

(1) Jinan Wang and Yinglong, M. Protein–Protein Interaction-Gaussian Accelerated Molecular Dynamics (PPI-GaMD): Characterization of Protein Binding Thermodynamics and Kinetics. *Journal of Chemical Theory and Computation* **2022**, *18*, 1275-1285. DOI: 10.1021/acs.jctc.1c00974.

(2) Vanommeslaeghe, K.; MacKerell, A. D. CHARMM additive and polarizable force fields for biophysics and computer-aided drug design. *Biochimica et Biophysica Acta (BBA) - General Subjects* **2015**, *1850* (5), 861-871. DOI: https://doi.org/10.1016/j.bbagen.2014.08.004.

(3) Duan, Y.; Wu, C.; Chowdhury, S.; Lee, M. C.; Xiong, G.; Zhang, W.; Yang, R.; Cieplak, P.; Luo, R.; Lee, T.; et al. A point-charge force field for molecular mechanics simulations of proteins based on condensed-phase quantum mechanical calculations. *Journal of Computational Chemistry* **2003**, *24* (16), 1999-2012. DOI: https://doi.org/10.1002/jcc.10349.

(4) Miao, Y.; Feher, V. A.; McCammon, J. A. Gaussian Accelerated Molecular Dynamics: Unconstrained Enhanced Sampling and Free Energy Calculation. *Journal of Chemical Theory and Computation* **2015**, *11* (8), 3584-3595. DOI: 10.1021/acs.jctc.5b00436.

(5) Miao, Y.; McCammon, J. A. Chapter Six - Gaussian Accelerated Molecular Dynamics: Theory, Implementation, and Applications. In *Annual Reports in Computational Chemistry*, Dixon, D. A. Ed.; Vol. 13; Elsevier, 2017; pp 231-278.

(6) Miao, Y. Acceleration of biomolecular kinetics in Gaussian accelerated molecular dynamics. *The Journal of Chemical Physics* **2018**, *149* (7), 072308. DOI: 10.1063/1.5024217 (acccessed 10/14/2025).
(7) Miao, Y.; Sinko, W.; Pierce, L.; Bucher, D.; Walker, R. C.; McCammon, J. A. Improved Reweighting of Accelerated Molecular Dynamics Simulations for Free Energy Calculation. *Journal of Chemical Theory and Computation* **2014**, *10* (7), 2677-2689. DOI: 10.1021/ct500090q.
(8) Valdés-Tresanco, M. S.; Valdés-Tresanco, M. E.; Valiente, P. A.; Moreno, E. gmx_MMPBSA: A New Tool to Perform End-State Free Energy Calculations with GROMACS. *Journal of Chemical Theory and Computation* **2021**, *17* (10), 6281-6291. DOI: 10.1021/acs.jctc.1c00645.
(9) Huang, J.; Rauscher, S.; Nawrocki, G.; Ran, T.; Feig, M.; de Groot, B. L.; Grubmüller, H.; MacKerell, A. D. CHARMM36m: an improved force field for folded and intrinsically disordered proteins. *Nature Methods* **2017**, *14* (1), 71-73. DOI: 10.1038/nmeth.4067.
